# Dysregulated lipid metabolites GML and GMO were associated with cytotoxic T cell function and serve as biomarkers for acute pulmonary embolism

**DOI:** 10.3389/fimmu.2026.1756977

**Published:** 2026-07-08

**Authors:** Tongyao Guo, Weibo Gao, Aimin Zhang, Dong Wang, Yushang Zhao, Yining Li, Zhihong Yue, Lin Pei, Mei Jia, Chen Liu, Lin-Lin Cao

**Affiliations:** 1 Department of Clinical Laboratory, Peking University People’s Hospital, Beijing, China; 2 Clinical Laboratory Center, Beijing Friendship Hospital, Capital Medical University, Beijing, China; 3 Department of Geriatrics, China-Japan Friendship Hospital, Beijing, China; 4 Application Support Center, Shanghai AB Sciex Analytical Instrument Trading Co., Ltd., Shanghai, China

**Keywords:** acute pulmonary embolism, cytotoxic T cells, glycerolipids, lipidomics, notch1 signaling

## Abstract

**Objective:**

To identify serum lipid biomarkers for acute pulmonary embolism (APE) and evaluate their diagnostic value, and to investigate the impact of glycerol monolaurate (GML) and glycerol monooleate (GMO) on cytotoxic T cell function.

**Methods:**

A total of 436 subjects, including APE, healthy controls, and patients with related diseases, were enrolled. Serum samples were subjected to pseudotargeted lipidomics to screen differential lipid species. Targeted LC-MS/MS analysis was used to validate the GML and GMO levels. Flow cytometry was used to assess cytotoxic T cell markers such as granzyme B, perforin, and granulysin. *In vitro* experiments examined the inhibitory effect of GML and GMO on Notch1 signaling and cytotoxic protein expression in T cells. ROC curve analyses were performed to evaluate the diagnostic performance of lipid biomarkers.

**Results:**

Pseudotargeted lipidomics identified 203 upregulated and 57 downregulated serum lipids in APE versus controls. Targeted LC-MS/MS confirmed significantly elevated serum GML and GMO in APE compared with healthy controls and other diseases. Serum GML and GMO were correlated with clinical risk stratification. ROC analyses showed high sensitivity and specificity for GML and GMO individually. Cytotoxic T cells from APE patients exhibited decreased granzyme B, perforin and granulysin. *In vitro*, GML and GMO reduced Notch1 and granzyme B expression in T cells.

**Conclusion:**

Elevated serum GML and GMO could serve as effective diagnostic biomarkers for APE, correlating with disease severity. They were likely to impair cytotoxic T cell function by downregulating Notch1 signaling and cytotoxic proteins, suggesting their involvement in APE pathogenesis.

## Introduction

Acute pulmonary embolism (APE) is a leading cause of cardiovascular death worldwide, with a mortality rate ranks third only to that of stroke and myocardial infarction (1). It is characterized by a high misdiagnosis, a high disability rates and poor clinical prognosis (2). The annual incidence of APE has exceeded 200,000 cases, with rates in China comparable to Europe and the US (3). Owing to its rapid onset, diverse and nonspecific clinical manifestations (4), APE is often misdiagnosed as non-ST-segment elevation myocardial infarction (NSTEMI), aortic dissection (AD), or pneumonia (5), leading to a 30%-50% rate of delayed diagnosis; the 30-day mortality of high-risk APE even reaches 15%-30%, posing a serious threat to patients’ lives (6). Therefore, it is necessary to explore new non-invasive biomarkers for the early diagnosis and severity assessment of APE to facilitate early intervention and personalized treatment, thereby improving patient outcomes.

APE diagnosis requires a multi-step approach combining clinical scoring, laboratory tests, and imaging examinations. Clinical scoring tools combined with D-dimer testing were served as preliminary screening methods for APE (7). While D-dimer testing serves as a valuable screening for ruling out APE, its low specificity necessitates confirmatory imaging (8). Arterial blood gas analysis and cardiac biomarkers, though helpful for assessment, lack sufficient specificity for standalone diagnosis (9). Imaging examinations are crucial for diagnosing APE, with computed tomographic pulmonary angiography (CTPA) being the gold standard due to its high sensitivity and specificity, but the patients were at risks of radiation exposure and contrast-induced nephropathy. Alternative methods such as ventilation/perfusion (V/Q) scanning, echocardiography, lower extremity venous ultrasound, and magnetic resonance pulmonary angiography (MRPA) offered different diagnostic approaches according to specific diagnostic needs and patient conditions, but these methods had certain limitations as well (10).

Metabolomics is a powerful platform for comprehensive small metabolites analysis, offering promising technical support for biomarkers discovery (11). APE is a complex cardiovascular disease related to thrombosis (7), involving numerous metabolic processes. Therefore, the occurrence of APE is bound to be accompanied by changes in the levels of various metabolites. Abnormal expression of various lipids, such as phospholipids, triglycerides, and long-chain fatty acids, has been reported in cardiovascular, metabolic, and inflammatory diseases, supporting their role in early diagnosis and targeted therapy (12, 13). Lipid metabolism was significantly associated with the occurrence and progression of APE. Elevated serum oxylipins, especially 20-hydroxy prostaglandin F2α (PGF2α) in the arachidonic acid pathway, have been identified in APE patients, suggesting their potential as diagnostic and therapeutic biomarkers (14). However, previous studies have only highlighted several lipid metabolites as diagnostic markers for APE, without comprehensive analysis of the lipidome and in-depth investigation on the underlying mechanisms. Therefore, it is essential to conduct a full-spectrum analysis of the lipidomic characteristics of APE patients and explore their underlying pathological mechanisms for the early diagnosis and effective management of APE.

Long-chain fatty acid (LCFA), which included saturated and unsaturated fatty acids with carbon chains exceeding 12, were not only significant metabolic substances within biological systems (15), but also occupied a central role in the exploration of pathogenesis, disease progression, and therapeutic strategies for a variety of conditions, including cardiovascular diseases (16, 17), neurological disorders (18, 19), and tumor-related diseases (20). Moreover, LCFAs had a significant impact on immune responses. Elaidic acid, a long-chain unsaturated fatty acid, played a crucial role in immune regulation by activating acyl-CoA Synthetase Long-Chain Family Member 5 (ACSL5), which in turn enhanced the major histocompatibility complex class I (MHC-I) antigen presentation in tumor cells. This process promoted the cytotoxic activity of CD8+ T cells, thereby suppressing tumor growth (21). Additionally, linoleic acid, another long-chain fatty acid, could also enhance the metabolic fitness of CD8+ T cells, which might help improve their antitumor activity (22).

In this study, we aimed to explore the dysregulated metabolites in APE compared to healthy control (HC), NSTEMI and AD individuals, and find potential biomarkers for early diagnosis and risk stratification of APE. Serum lipid profiles in HC and APE patients were determined by pseudotargeted lipidomic analysis. The lipids that were differentially expressed in APE compared with HC, NSTEMI and AD individuals were validated by targeted metabolite analyses. The diagnostic value and clinical correlations of these biomarkers were also evaluated. In addition, how these dysregulated lipids exerted their function in APE and the underlying mechanisms were determined as well. Overall, our study was the first to explore candidate lipid biomarkers for the diagnosis of APE by pseudotargeted lipidomics, and investigate their potential role in disease progression.

## Materials and methods

### Chemicals and reagents

Standards and Internal Standard (IS) solution for 1-linoleoyl-rac-glycerol (GML) and rac 1-oleoyl glycerol (GMO) were all obtained from Zhenzhun Biotechnology Co., Ltd. (Shanghai, China). Liquid chromatography grade methanol was purchased from Thermo Fisher Scientific Inc. (Waltham, USA). Deionized water was obtained from Watsons (Guangzhou, China). The antibodies utilized in this research were obtained from Biolegend (San Diego, CA, USA). The Notch1 signaling agonist, sodium valproate (VPA), was purchased from MedChemExpress (MCE) Company (Monmouth Junction, USA).

### Study population

**Figure 1:**
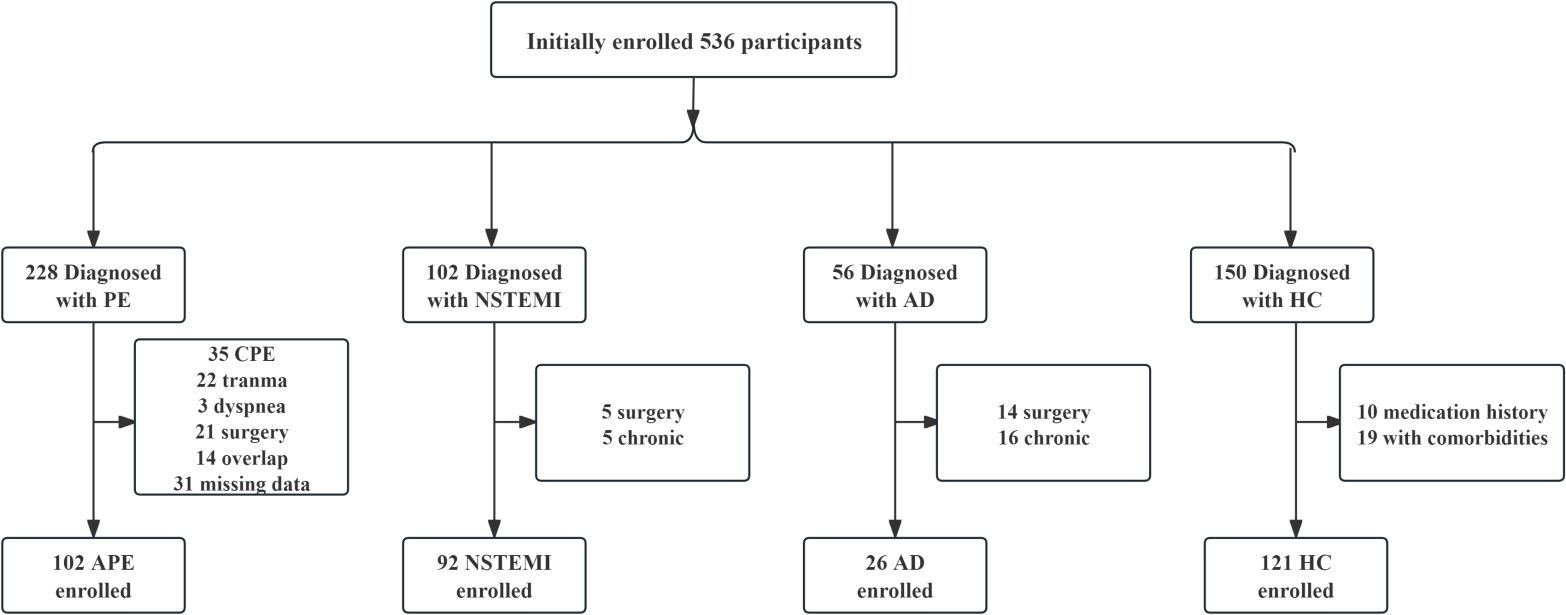
Patient selection flowchart. HC, healthy control; APE, acute pulmonary embolism; CPE, chronic pulmonary embolism; NSTEMI, non-ST-segment elevation myocardial infarction; AD, aortic dissection. There was no overlap between the diagnostic categories of these diseases.

A total of 501 individuals were recruited between March 2023 and October 2024 from Peking University People’s Hospital (Beijing, China) in this study, including HCs, patients with APE, NSTEMI, AD, and chronic pulmonary embolism (CPE, also referred to as chronic pulmonary thromboembolism, CPTE). Based on the inclusion and exclusion criteria for each disease, a total of 436 participants were ultimately enrolled (**Figure 1**). All individuals were divided into a discovery cohort and a validation cohort. The discovery cohort included 30 HC and 30 APE patients for pseudotargeted lipidomic analysis. The validation cohort included 121 HCs, 102 APE, 92 NSTEMI, 26 AD, and 35 CPE patients for targeted metabolite analysis and validation of the diagnostic performance of differential lipids. Among them, 23 HCs and 20 APE patients were used for flow cytometry analysis to evaluate the cytotoxic function of T cells in APE patients. Sample size calculation was performed using G*Power 3.1 software. For the discovery cohort, based on an expected effect size of 0.8 for differential lipid metabolites between APE and HC groups, with a two-sided α of 0.05 and power of 0.80, a minimum of 26 subjects per group was required (23–25). We enrolled 30 subjects per group to account for potential dropouts. For the validation cohort, the sample size was expanded to ensure adequate power (>90%) for validating the diagnostic performance of GML and GMO across multiple comparison groups. HCs were individuals who appeared healthy and underwent routine medical check-ups in Peking University People’s Hospital. The inclusion criteria for HCs patients were: (1) Age ≥ 18 years; (2) No history of acute or chronic thromboembolism, no cardiovascular or cerebrovascular diseases, no malignancy, autoimmune disease, active infection, or severe hepatic, renal or hematologic disorders; (3) No history of special medication use. The exclusion criteria were: (1) Pregnant or lactating women; (2) History of smoking or alcohol consumption; (3) Any acute or chronic diseases that may affect lipid metabolism or immune function. The diagnosis of APE patients was in accordance with the “2019 ESC Guidelines for the Diagnosis and Management of Acute Pulmonary Embolism” published by the European Society of Cardiology (26). The inclusion criteria for APE patients were: (1) Age ≥ 18 years; (2) Patients confirmed with pulmonary thromboembolism by CTPA; (3) Patients with a first-time diagnosis of pulmonary thromboembolism; (4) Patients with complete clinical data. The exclusion criteria were: (1) History of previous pulmonary embolism; (2) Patients who had received anticoagulant or thrombolytic therapy prior to the onset of illness. For CPE patients, the inclusion criteria were: (1) Age ≥18 years; (2) Presence of symptoms such as exertional dyspnea, fatigue, and chest pain after at least three months of standardized anticoagulant therapy; (3) V/Q scan indicating perfusion defects in lung lobes and/or segments that do not match ventilation, or chronic thromboembolic signs (such as organized thrombi, band-like stenoses, and web-or-sling lesions) in the pulmonary arteries as shown by CTPA; (4) Echocardiography suggesting the possibility of pulmonary arterial hypertension (such as tricuspid regurgitant velocity > 2.8 m/s and estimated systolic pulmonary arterial pressure≥40 mmHg). The exclusion criteria for CPE were: (1) Patients have not undergone at least three months of standardized anticoagulant therapy; (2) Negative V/Q scan or CTPA does not show typical signs of chronic thromboembolism; (3) Exclusion of pulmonary arterial hypertension caused by other diseases (such as left-heart diseases, primary pulmonary arterial hypertension, lung diseases, congenital heart diseases, etc.). The inclusion criteria for NSTEMI were: (1) Age ≥18 years; (2) Patients were clearly diagnosed with NSTEMI in accordance with the Guidelines for the management of non-ST elevation acute coronary syndromes (2024 version); (3) Patients with a first-time diagnosis of NSTEMI; (4) Patients with complete clinical information. The exclusion criteria for NSTEMI were: (1) Two consecutive high-sensitivity cardiac troponin tests show results within the normal range, and the change in values between the two tests does not meet diagnostic criteria; (2) Electrocardiogram (ECG) does not show ischemic changes such as ST-segment depression, and there are no dynamic changes; (3) Clinical symptoms are atypical and without obvious precipitating factors. For AD patients, the inclusion criteria were: (1) Age ≥ 18 years; (2) Patients confirmed with aortic dissection by computed tomography angiography (CTA) or magnetic resonance angiography (MRA); (3) Patients with a first-time diagnosis of aortic dissection; (4) Patients with complete clinical data. The exclusion criteria for AD were: (1) Within 24 hours of symptom onset, patients with no significant elevation in D-dimer levels; (2) Patients whose CTA or MRA did not show signs of dissection. Serum samples from APE patients were collected within 24 hours of symptom onset and prior to initiation of anticoagulant or thrombolytic therapy. Samples from NSTEMI, AD, and CPE patients were collected within 24 hours of definitive diagnosis. Healthy control samples were obtained during routine medical check-ups. This study was conducted in accordance with the principles of the Helsinki Declaration and approved by the Ethical Review Committee of Peking University People’s Hospital (2024PHB059-001).

### Measurement of clinical indicators

Complete blood cell counts (CBC) were ascertained through the Sysmex XN-9000 (Sysmex, Kobe, Japan). The coagulation function-related parameters, including prothrombin time (PT), activated partial thromboplastin time (APTT), fibrinogen, and D-dimer, were determined using ACL TOP 9000 (Werfen, Barcelona, Spain). Serum alanine aminotransferase (ALT), aspartate aminotransferase (AST), lactate dehydrogenase (LDH), albumin (ALB), blood urea nitrogen (BUN), creatinine (CRE), total cholesterol (TCHO), triglycerides (TG), high-density lipoprotein cholesterol (HDL-C) and low-density lipoprotein cholesterol (LDL-C) were detected by Beckman AU5800 (Beckman Coulter, Inc., USA). All measurements were performed using reagents that had undergone rigorous method validation, strictly adhering to the manufacturer’s standard operating procedures. Comprehensive internal quality control and external quality assessment were implemented to ensure the accuracy and precision of the test results.

### Pseudotargeted lipidomic analyses

After centrifugation of peripheral blood, 1 mL serum was transferred to a preservative tube and immediately stored at −80 °C until analysis. Before analysis, serum samples were thawed completely at room temperature, vortexed for 10 s to mix thoroughly, and then 50 μL of the sample was transferred to a centrifuge tube. Then 1mL of the lipid extraction solution containing the internal standard (methyl tert-butyl ether: methanol = 3:1, V/V) was added, followed by vortexing for 15 min. Subsequently, 200 μL of water was added, and the mixture was vortexed for 1 min, then centrifuged at 12,000 r/min for 10 min at 4°C. The supernatant (200 μL) was transferred to a centrifuge tube and evaporated to complete dryness. The residue was reconstituted with 200 μL of the lipid reconstitution solution (acetonitrile: isopropanol = 1:1, V/V), vortexed for 3 min, and then centrifuged at 12,000 r/min for 3 min at 4 °C. The supernatant was collected for LC-MS/MS analysis.

### Targeted metabolite analyses

The standard curve was prepared by serially diluting the stock solution 2-fold in 50% methanol, and obtained 6 calibration points. Meanwhile, the IS working solution was prepared by mixing equal volumes of the two internal standard stock solutions (GML-D5 and GMO-D5). In addition, the quality control (QC) samples (low-level, medium-level and high-level) were prepared by spiking appropriate amounts of the standards of GML and GMO into a mixed serum. Subsequently, the pretreatment of calibrators, QC and serum samples was performed according to the following procedure. 100 μL of sample and 400 μL of precipitant (100% methanol) containing IS were added into a 96-well microplate. The mixture was then vortexed for 2 min and centrifuged at 4,000 rpm for 15 min. The supernatant was then transferred into another 96-well plate, centrifuged at 4,000 rpm for 15 min again. The supernatant was subjected to LC-MS/MS analysis.

The LC-MS/MS analysis was conducted using the Triple Quad™ 4500MD mass spectrometer (AB SCIEX, Foster City, USA). The chromatographic column used was a Kinetex C18 column (2.1 mm×100 mm, 2.6 μm) (Phenomenex Corporation, USA), using 0.1% formic acid water solution as the mobile phase A and 0.1% formic acid methanol solution as mobile phase B, at the flow rate was 0.3 mL/min. The column temperature was set at 40 °C, and the auto-sampler temperature was set at 4 °C. The total run time for each sample was 7 min, and the gradient elution program was shown in **Supplementary Table 1**. Data acquisition was performed in positive electrospray ionization (ESI+) mode. The MS/MS parameters were optimized, and the final multiple reaction monitoring (MRM) transitions were shown in **Supplementary Table 2**. The ion spray voltage was set at 5500 V. The optimal probe temperature was 400 °C. The nebulizer gas (Gas 1) was set at 50 psi, the heater gas (Gas 2) at 45 psi, the curtain gas at 20 psi, and the collision gas at 8 psi. The MS/MS spectra were generated using Analyst MD 1.6.3 software (AB SCIEX, Foster City, USA). Quantification was performed with MultiQuant MD 3.0.3 software (AB SCIEX, Foster City, USA).

### Flow cytometry

Peripheral blood mononuclear cells (PBMCs) were isolated using Ficoll-Paque density gradient centrifugation (Dakewe Biotech Co., Ltd., China). The surface marker staining was performed with fluorochrome-conjugated antibodies (BioLegend, USA). Cells were incubated with the antibody cocktail at 2-8 °C in the dark for 30 min. After surface staining, cells were fixed, permeabilized, and stained for intracellular markers using fluorescent antibodies (BioLegend, USA). Gating thresholds for positive and negative populations were determined based on fluorescence-minus-one (FMO) controls. Following staining, cells were washed twice and analyzed on a FACSCanto™ flow cytometer using Diva software (BD Biosciences, San Jose, CA, USA). Two antibody panels were used for flow cytometry analysis: Panel 1 included anti-CD4-APC-Cy7, anti-CD8-APC, anti-GZMB-PerCP-Cy5.5, anti-perforin-FITC, and anti-granulysin-PE. Panel 2 consisted of anti-CD8-APC-Cy7, anti-CD4-APC, anti-CD45RA-PE-Cy7, anti-GZMB-PerCP-Cy5.5, anti-Emoes-FITC, and anti-Notch1-PE.

### Cell culture

6 healthy controls (HCs) were recruited from Peking University People’s Hospital. Peripheral blood mononuclear cells (PBMCs) were isolated, and each sample of PBMCs was divided into 12 portions. Each aliquot was cultured in RPMI 1640 medium supplemented with 10% fetal bovine serum. Different concentrations of GML, GMO, and VPA were added to the medium. The cells were incubated at 37 °C in a 5% CO2 atmosphere for 24 hours, followed by flow cytometry analysis.

### Statistical analysis

Statistical analyses were performed using SPSS 27.0 (SPSS Inc., Chicago, USA) and GraphPad Prism 10.0 (GraphPad Corporation, La Jolla, CA, USA). For comparisons between two groups, normally distributed data were analyzed using the student’s *t* test, while non-normally distributed data were analyzed using the Mann-Whitney *U* test. For comparisons among three or more groups, one-way ANOVA was used for normally distributed data, and the Kruskal-Wallis test for non-normally distributed data. Spearman correlation analysis was employed to determine the correlation coefficient (*r*) and the corresponding *P* value. To address potential confounding by baseline characteristics, multivariable logistic regression was performed adjusting for clinical covariates. Additionally, to evaluate the diagnostic efficacy of long-chain fatty acids for APE, receiver operating characteristic (ROC) curve analysis was conducted, and the area under the curve (AUC) was calculated. A combined diagnostic model incorporating both GML and GMO was developed using binary logistic regression. The logistic equation was: Logit(P) =β_0_+β_1_×[GML] +β_2_×[GMO], whereβ_0_ represents the intercept, andβ_1_ andβ_2_ represent the regression coefficients for GML and GMO, respectively. All statistical tests were two-tailed, with *P* < 0.05 considered statistically significant. Differential metabolites in the discovery phase were defined using the following criteria: fold change (FC) > 1.5 or < 0.67, variable importance in projection (VIP) > 1, and nominal P < 0.05. Given the exploratory nature of the discovery-phase lipidomics screening, nominal P-values were used to prioritize potential candidates for subsequent validation, with the understanding that findings would be confirmed in an independent validation cohort using targeted LC-MS/MS.

## Results

### Pseudotargeted lipidomic profiling identified significantly dysregulated serum lipids in APE patients

**Figure 2:**
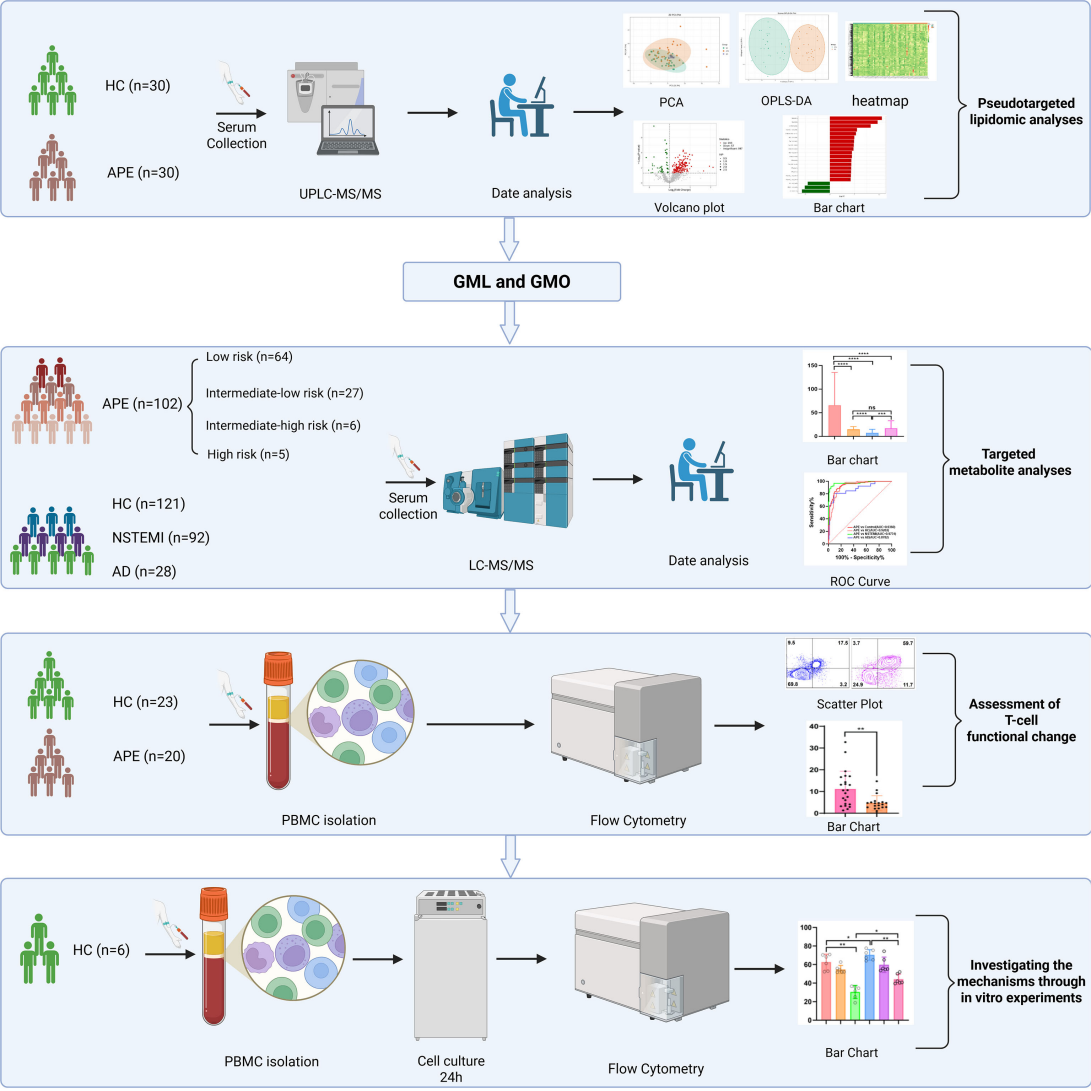
The flow diagram of the study.

**Figure 3:**
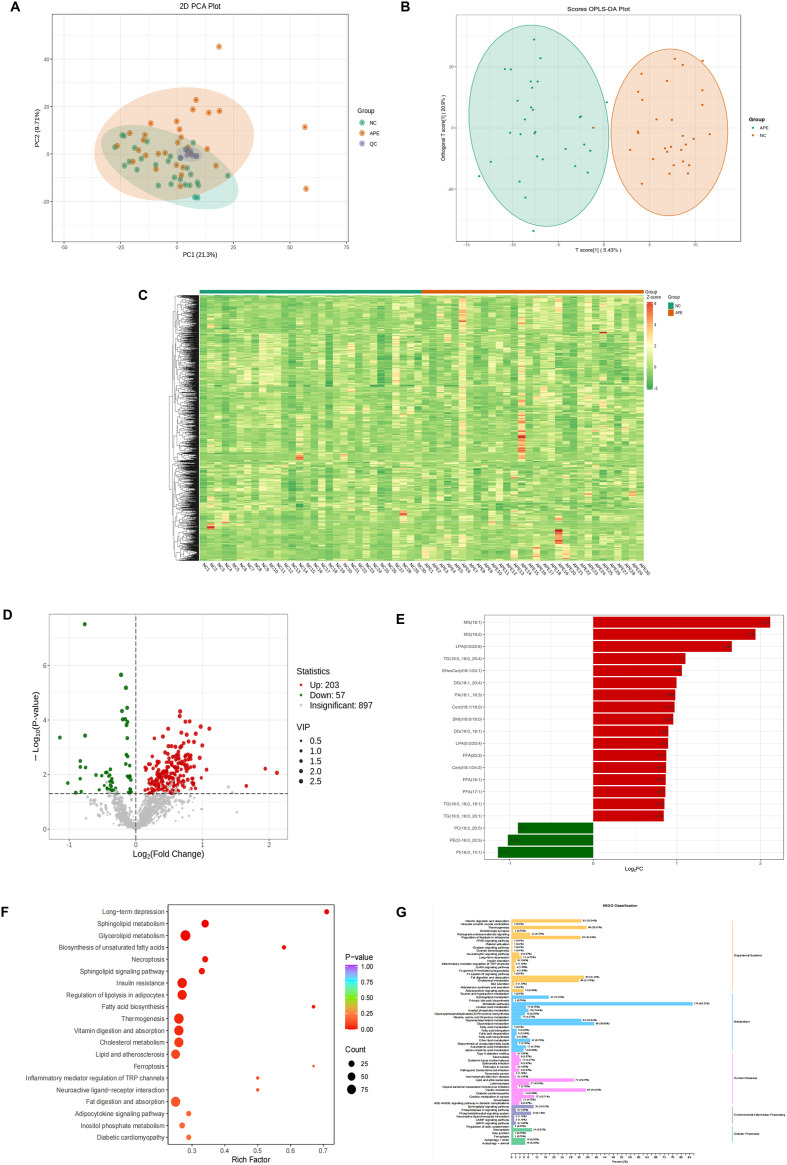
Pseudotargeted lipidomic profiling of serum samples from APE patients and HC individuals in the discovery cohort. **(A)** The principal component analysis (PCA) score plot. **(B)** Score plot of orthogonal projections to latent structures-discriminate analysis (OPLS-DA). **(C)** Hierarchical clustering analysis revealed differential lipid metabolites between the two groups. Each column represented a sample, and each row represented a metabolite. Red indicated high levels, and green indicated low levels. **(D)** The volcano plot displayed the differential lipid metabolites between HC and APE groups. Red points referred to significantly upregulated metabolites and green points referred to significantly downregulated ones according to fold change > 1.5 or < 0.67, VIP > 1 and nominal P < 0.05. **(E)** This bar chart illustrated and ranked the most significantly differential lipid metabolites between the HC and APE groups. The red bars represented significantly upregulated metabolites and green bars showed downregulated ones. Longer bars indicated a larger fold change. **(F)** KEGG pathway enrichment analysis of differential lipids. Enrichment analysis was performed based on differentially expressed lipids identified in the discovery cohort (n=260, APE vs. healthy controls, FC > 1.5 or < 0.67, VIP > 1, P < 0.05). The x-axis represents the Rich Factor. The y-axis lists significantly enriched KEGG pathways. Bubble size indicates the number of differentially expressed lipids mapped to each pathway, and color intensity represents statistical significance (red: P < 0.05; orange: P ≥ 0.05). **(G)** Functional classification of enriched KEGG pathways. The bar chart illustrates the distribution of differential lipids across biological functional categories. The x-axis indicates the percentage of differential lipids (%) mapped to each pathway. Colors denote functional categories: Metabolism (blue), Human Diseases (pink), Environmental Information Processing (purple), Cellular Processes (green), and Organismal Systems (yellow).

The workflow scheme of the study was presented in **Figure 2**, and the demographic and clinical characteristics of individuals included in the discovery and validation cohort were shown in **Table 1**. In the discovery cohort, pseudotargeted lipidomic analyses were conducted on serum samples from individuals in HC and APE groups. The quality control (QC) points nearly coincided in the principal component analysis (PCA) plot (**Figure 3A**), indicating the analytical method had high repeatability and stability. In addition, the robustness of the assay was indicated by the extremely strong correlation observed among the QC samples (**Supplementary Figure 1**). Subsequently, the orthogonal projections to latent structures-discriminate analysis (OPLS-DA) model revealed a clear separation between HC and APE groups (**Figure 3B**). The cumulative R^2^Y and Q^2^ was 0.837 and 0.56, respectively (p < 0.005), indicating that the model had outstanding predictive capability, with no signs of overfitting observed (**Supplementary Figure 2**). The results of hierarchical cluster analysis revealed a very significant difference in the lipid profile between two groups (**Figure 3C**). The serum lipids with significant differences between APE and HC groups were shown in the volcano plot, with a total of 203 upregulated and 57 downregulated lipids were identified based on the criteria of FC > 1.5 or < 0.67, VIP > 1, and nominal P < 0.05 (**Figure 3D**; **Supplementary Table 3**). Subsequently, the bar chart displayed that two long-chain fatty acids, 1-linoleoyl-rac-glycerol (GML) and rac 1-oleoyl glycerol (GMO), were the most differentially upregulated lipid metabolites in APE (**Figure 3E**). Furthermore, KEGG pathway enrichment analysis revealed significant enrichment of glycerolipid metabolism (encompassing GML and GMO), sphingolipid metabolism, and lipid and atherosclerosis pathways (**Figure 3F**). Class-level analysis further demonstrated that 85.32% of differential lipids mapped to metabolic pathways (**Figure 3G**), indicating systematic lipid network reprogramming centered on glycerolipid and sphingolipid metabolism in APE. GML was an ester compound formed by the esterification of linoleic acid with glycerol, and could also be represented as MG(18:2). Similarly, GMO was an ester compound generated by the reaction of oleic acid with glycerol, and could also be represented as MG(18:1).

**Table 1:** The demographic and clinical characteristics of individuals included in this study.

Variables	Discovery cohort			Validation cohort					
	HC	APE	*P-*value	HC	APE	NSTEMI	AD	CPE	*P-*value
	n=30	n=30		n=121	n=102	n=92	n=26	n=35	
Age	56.13 ± 16.23	57.70 ± 16.19	0.5791	63.70 ± 9.90	65.70 ± 17.00	66.60 ± 14.60	62.42 ± 10.36	66.20±14.50	0.228
Gender Male/Female	13/17	13/17	>0.9999	67/54	58/44	62/30	22/4	14/21	0.106
WBC (×10^9^/L)	–	–		5.16 (2.27)	7.88 (3.85)^****^	8.60 (3.98)^****^	10.14 (7.22)^****^	5.70 (2.07)^NS^	<0.0001
N (%)	–	–		57.51 ± 8.86	69.53±12.34^****^	70.66±13.41^****^	83.33±9.04^****^	54.83±11.41^NS^	<0.0001
Lym (%)	–	–		33.30 (11.70)	21.70 (16.80)^****^	18.90 (16.60)^****^	6.40 (10.21)^****^	36.60 (10.05)^NS^	<0.0001
PLT (×10^9^/L)	–	–		220.00 (65.00)	194.00 (101.00)^*^	193.00 (100.50)^**^	188.50 (146.20)^NS^	219.00 (116.00)^NS^	0.0009
PT (s)	–	–		11.00 (0.85)	12.25 (2.78)^****^	11.45 (1.68)^**^	12.20 (1.95)^**^	11.50 (3.50)^*^	<0.0001
APTT (s)	–	–		30.40 (3.60)	30.80 (6.35)^NS^	31.00 (4.88)^NS^	29.45 (8.15)^NS^	31.65 (4.25)^NS^	0.5466
FIB (mg/dL)	–	–		316.00 (52.50)	343.00 (115.00)^NS^	361.00 (146.30)^*^	360.00 (140.20)^NS^	327.00 (71.20)^NS^	0.0223
FDP (μg/mL)	–	–		1.00 (0.20)	9.50 (18.73)^****^	2.00 (3.00)^NS^	17.00 (22.33)^****^	1.10 (3.23)^NS^	<0.0001
D-D (ng/mL)	–	–		130.00 (148.00)	1225.00 (2807.00)^****^	285.00 (512.00)^NS^	2252.00 (2730.70)^****^	119.00 (181.50)^NS^	<0.0001
ALT (U/L)	–	–		16.00 (10.00)	19.00 (20.00)^*^	21.00 (17.00)^***^	20.50 (40.25)^NS^	18.00 (8.50)^NS^	0.0016
AST (U/L)	–	–		21.00 (7.00)	21.00 (13.00)^NS^	34.00 (42.00)^****^	26.00 (48.25)^NS^	26.00 (11.50)^NS^	<0.0001
LDH (U/L)	–	–		181.00 (33.80)	221.00 (107.00)^****^	260.00 (150.00)^****^	292.50 (289.50)^****^	198.00 (57.50)^NS^	<0.0001
ALB (g/L)	–	–		44.57±2.37	39.34±6.68^****^	39.09±5.10^****^	37.37±5.44^****^	44.35±3.68^NS^	<0.0001
TBIL (μmol/L)	–	–		15.40 (7.50)	10.50 (7.30)^****^	11.90 (8.00)^*^	17.90 (24.50)^NS^	11.60 (7.35)^*^	<0.0001
DBIL (μmol/L)	–	–		4.30 (2.23)	4.00 (2.80)^NS^	4.40 (3.90)^NS^	6.95 (12.88)^*^	3.80 (2.25)^NS^	0.0008
Urea (mmol/L)	–	–		4.90 (1.70)	5.60 (2.88)^*^	7.10 (4.50)^****^	8.20 (7.10)^***^	5.40 (2.62)^NS^	<0.0001
CRE (μmol/L)	–	–		67.00 (17.75)	74.50 (34.75)^**^	84.00 (62.00)^****^	94.00 (62.30)^***^	73.00 (22.35)^NS^	<0.0001
CHO (mmol/L)	–	–		4.88 ± 0.90	4.54 ± 1.20^NS^	4.18 ± 1.18^****^	3.91 ± 1.23^**^	4.69 ± 0.98^NS^	<0.0001
TG (mmol/L)	–	–		1.01 (0.64)	1.49 (0.74)^****^	1.01 (0.37)^NS^	1.26 (0.68)^NS^	1.35 (0.80)^**^	<0.0001
HDL (mmol/L)	–	–		1.51 ± 0.30	1.18 ± 0.39^****^	1.03 ± 0.29^****^	1.20 ± 0.46^**^	1.47 ± 0.41^NS^	<0.0001
LDL (mmol/L)	–	–		2.89 ± 0.74	2.76 ± 0.98^NS^	2.48 ± 0.96^**^	2.23 ± 1.05^*^	2.57 ± 0.78^NS^	0.0036

The APE, NSTEMI, AD, and CPE groups were compared with the HC group. N (%), Neutrophil Percentage; Lym (%), Lymphocyte Percentage; D-D, D-dimer; NS, no significant; ^*^P<0.05; ^**^P<0.01; ^***^P<0.001; ^****^P<0.0001.

### Method validation of a novel LC-MS/MS method for detecting GML and GMO

To validate the results of the pseudotargeted lipidomic analysis and explore the diagnostic performance of serum GML and GMO for APE, a novel targeted LC-MS/MS method was developed for the quantitative determination of GML and GMO in human serum. The method was described in detail in the Materials and Methods, and method validation was carried out to assess the linear range, sensitivity, carryover, precision, accuracy, and matrix effect of the LC-MS/MS approach for detecting GML and GMO. The chromatograms of a representative serum sample were shown in **Supplementary Figure 3**. The linear correlation coefficients for the calibration curves of GML and GMO were >0.99 (**Supplementary Figure 4**), and the linear range and the limit of quantification (LoQ) of each analyte were shown in **Table 2**. Rigorous quality control was conducted on low-level quality control (LQC), medium-level quality control (MQC) and high-level quality control (HQC) samples. Intra- and inter-day precisions were assessed by running 3 replicates of 3 QC levels each day for three consecutive days, and the intra-day and inter-day imprecisions were acceptable with all coefficients of variation (CVs) <10%. No significant carryover was detected for the analytes when a blank sample was tested immediately following the highest calibration standard (**Supplementary Figure 5**). In addition, recovery was evaluated by spiking serum samples with known low, medium and high concentrations of standard solutions in triplicate, and the results were satisfactory with recovery rate between 90% and 110%. The matrix effect was evaluated by preparing three solutions: (A) serum sample with low/medium/high concentration standards, (B) blank serum sample, and (C) pure solution (1% BSA) with low/medium/high concentration standards. The matrix effect (%) was calculated using the formula [(A - B)/C] × 100%, and the results ranged from 90% to 110%, indicating the high accuracy of the LC-MS/MS method (**Table 2**).

**Table 2:** The results of methodology validation for the LC-MS/MS method.

Parameters		Level/Type	GML	GMO
Precisions (%)	intra-day	Low	1.29	0.98
		Medium	1.46	0.30
		High	0.90	2.22
	inter-day	Low	8.51	10.00
		Medium	6.42	6.59
		High	3.45	5.15
Linear Range (μg/mL)			5-200	5-200
Limit of Detection (LOD, μg/mL)			1	1
Limit of Quantification (LOQ, μg/mL)			5	5
Regression Coefficient (R^2^)			0.9992	0.9940
Recoveries (%)	Low		103.92	107.41
	Medium		93.57	92.17
	High		106.06	106.21
Matrix Effect (%)	Low		91.66	95.97
	Medium		96.41	95.23
	High		109.05	100.74

### Validation of the dysregulated lipids GML and GMO by targeted metabolite analyses

**Figure 4:**
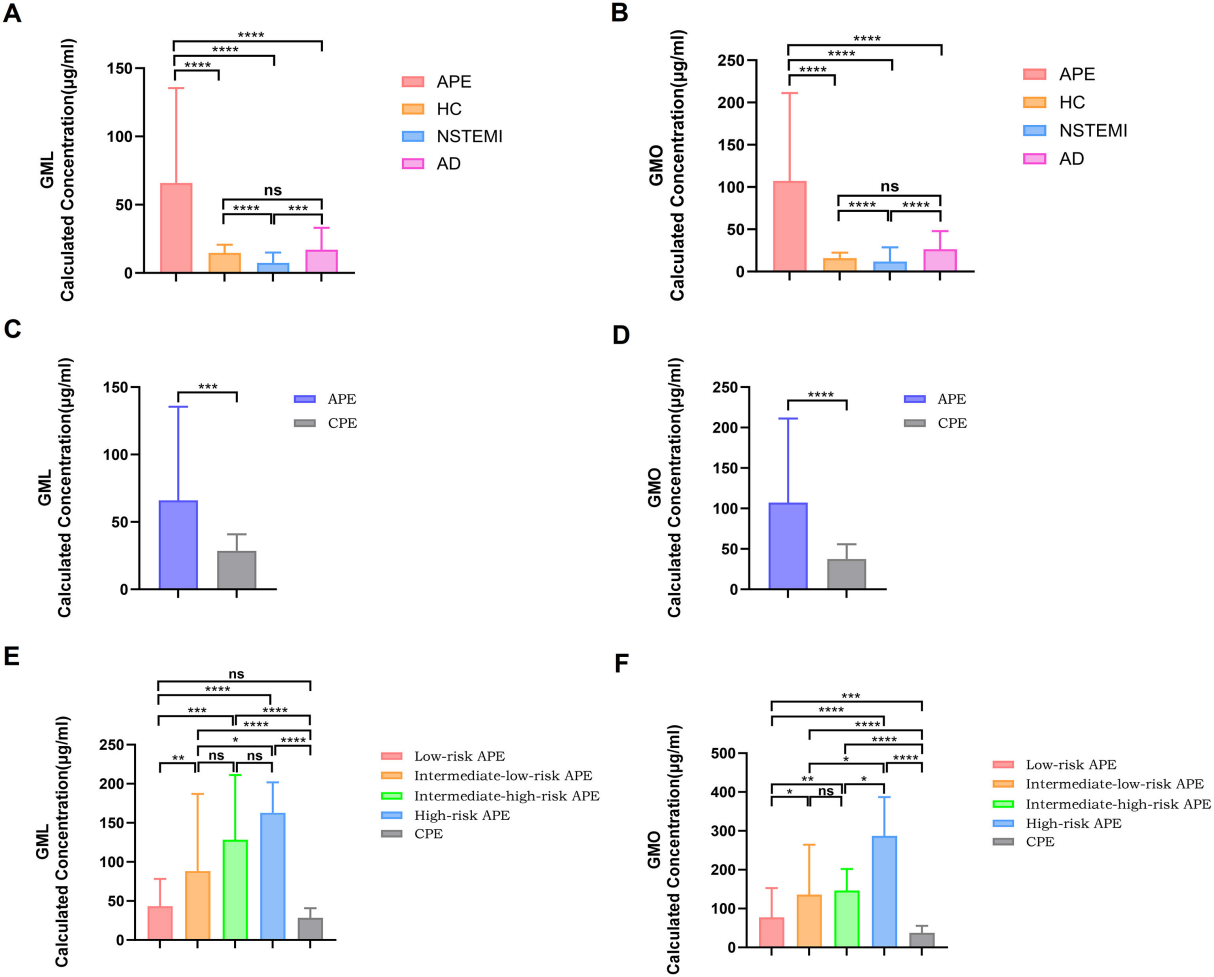
Expression profiles of serum GML and GMO in the validation cohort. **(A)** The bar chart depicted the levels of serum GML (μg/ml) among four groups: APE (n=102), HC (n=121), NSTEMI (n=92), and AD (n=26). **(B)** The bar chart depicted the levels of serum GMO (μg/ml) among four groups: APE (n=102), HC (n=121), NSTEMI (n=92), and AD (n=26). **(C)** The bar chart depicted the levels of GML (μg/ml) in APE (n=102) and CPE (n=35). **(D)** The bar chart depicted the levels of GMO (μg/ml) in APE (n=102) and CPE (n=35). **(E)** This bar chart depicted the levels of GML (μg/ml) across different risk stratifications (low risk [n=64], intermediate-low risk [n=27], intermediate-high risk [n=6], high risk [n=5]) within the APE group (n=102). **(F)** This bar chart depicted the levels of GMO (μg/ml) across different risk stratifications (low risk [n=64], intermediate-low risk [n=27], intermediate-high risk [n=6], high risk [n=5]) within the APE group (n=102). ns, not significant; *P<0.05; **P<0.01; ***P<0.001; ****P<0.0001.

To further validate the diagnostic value of GML and GMO for APE patients, their expression levels in the validation cohort were detected using the above targeted LC-MS/MS method. Consistent with the discovery cohort, APE patients had significantly higher levels of GML and GMO compared to HC group. Surprisingly, APE patients also had higher levels of GML and GMO than NSTEMI and AD patients, suggesting that GML and GMO were promising diagnostic biomarkers with excellent specificity for APE (**Figures 4A, B**). Meanwhile, the serum concentrations of GML and GMO in HC and AD groups were higher than those in NSTEMI patients, while there was no significant difference between HC and AD groups. Additionally, compared to the CPE patients, the levels of GML and GMO in the serum of APE patients were significantly elevated (**Figures 4C, D**). These results suggested that GML and GMO were specifically upregulated in the serum of APE patients.

### Significant variations in GML and GMO levels among APE patients based on risk stratification

According to the “Guidelines for the Diagnosis and Management of Acute Pulmonary Embolism” published by the European Society of Cardiology (ESC) in 2019, the risk stratification for APE could be categorized into low risk, intermediate-low risk, intermediate-high risk, and high risk. Then we determined the significance of GML and GMO in risk assessment for APE patients by comparing their levels across various risk stratification. The results indicated that the serum GML concentrations in patients with APE in the intermediate-low-risk, intermediate-high-risk, and high-risk groups were significantly higher than those in the low-risk group and the CPE group, with no significant difference observed between the CPE patients and the low-risk group. The serum GML concentrations in the high-risk group of APE patients were significantly higher than those in the intermediate-low-risk group, while there was no significant difference between intermediate-high-risk patients and intermediate-low-risk, as well as high-risk patients (**Figure 4E**). Regarding the serum GMO concentrations in APE patients across different risk states, the concentrations in the intermediate-low, intermediate-high, and high-risk groups were significantly higher than those in the low-risk group, and the CPE patients had significantly lower concentrations than the low-risk group. The serum GMO concentrations in the high-risk group of APE patients were significantly higher than those in the intermediate-low-risk and the intermediate-high-risk group, but there were no significant differences between the intermediate-low-risk group and the intermediate-high-risk group (**Figure 4F**). The levels of GML and GMO gradually increased from the intermediate-low-risk group to the intermediate-high-risk group, and then to the high-risk group, although some statistical p-values were greater than 0.05. These results suggested significant differences in the concentrations of the two long-chain fatty acids, GML and GMO, among APE patients with different risk stratifications, indicating that GML and GMO may serve as promising biomarkers for assessing the disease condition of APE patients and potentially be used to predict the prognosis of APE patients.

### Evaluation of the diagnostic performances of GML and GMO as novel biomarkers for APE

**Figure 5:**
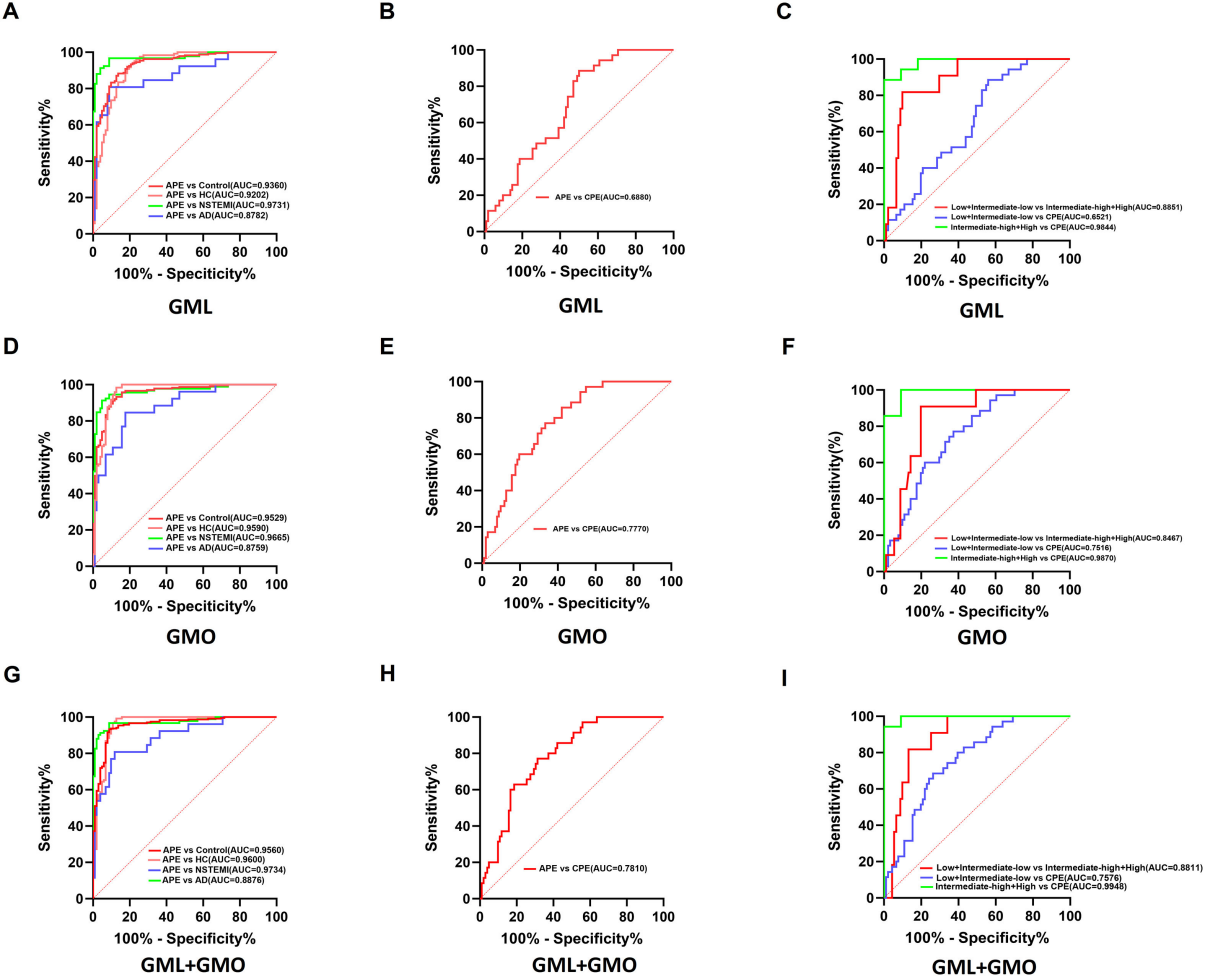
Assessing the diagnostic performance of GML and GMO as novel biomarkers for APE in the validation cohort. **(A)** The ROC curve of serum GML for discriminating APE (n=102) patients from Control (combination of HC, NSTEMI, and AD groups [n=239]), HC (n=121), NSTEMI (n=92), and AD (n=26). **(B)** The ROC curve of serum GML for discriminating APE (n=102) patients from CPE (n=35). **(C)** The ROC curve of serum GML for discriminating different risk stratifications (low risk [n=64], intermediate-low risk [n=27], intermediate-high risk [n=6], high risk [n=5]) in APE patients. **(D)** The ROC curve of serum GMO for discriminating APE (n=102) patients from Control (combination of HC, NSTEMI, and AD groups [n=239]), HC (n=121), NSTEMI (n=92) and AD (n=26). **(E)** The ROC curve of serum GMO for discriminating APE (n=102) patients from CPE (n=35). **(F)** The ROC curve of serum GMO for discriminating different risk stratifications (combination of HC, NSTEMI, and AD groups [n=239]) in APE patients. **(G)** The ROC curves for the combination of serum GML and GMO in discriminating APE (n=102) patients from Control (combination of HC, NSTEMI, and AD groups [n=239]), HC (n=121), NSTEMI (n=92) and AD (n=26). **(H)** The ROC curves for the combination of serum GML and GMO in discriminating APE (n=102) patients from CPE (n=35). **(I)** The ROC curves for the combination of serum GML and GMO for discriminating different risk stratifications (combination of HC, NSTEMI, and AD groups [n=239]) in APE patients.

Then we evaluated the diagnostic potential of GML and GMO for APE using ROC curves. As depicted in **Figure 5A** and **Table 3**, GML demonstrated AUC values of 0.9360, 0.9202, 0.9731, 0.8782 in differentiating APE patients from Control (combined of HC, NSTEMI, and AD groups), HC, NSTEMI, and AD groups, respectively. The corresponding optimal sensitivities were 88.28-91.30%, and specificities were 81.37-96.08%. For discriminating APE from CPE, GML exhibited an AUC of 0.6880, with a sensitivity of 88.57% and specificity of 50.00% (**Figure 5B**; **Table 3**). Furthermore, for discriminating low-risk and intermediate-low-risk APE from intermediate-high-risk and high-risk APE, GML displayed an AUC of 0.8851, with sensitivity and specificity of 81.82% and 90.11%, respectively. Meanwhile, GML revealed an AUC of 0.6521 in discriminating low-risk and intermediate-low-risk APE from CPE, and the optimal sensitivity and specificity were 88.57% and 43.96%, respectively. In addition, it revealed an AUC of 0.9844 in discriminating intermediate-high-risk and high-risk APE from CPE, with the optimal sensitivity and specificity of 88.57% and 100%, respectively. (**Figure 5C**; **Table 3**).

**Table 3:** Diagnostic value of GML and GMO for the early diagnosis of APE.

Biomarker	Comparison	AUC	Cutoff (μg/mL)	Sensitivity (%)	Specificity (%)	P value	95%CI
GML	APE vs Control	0.9360	20.69	88.28	86.27	<0.0001	0.9086-0.9634
	APE vs HC	0.9202	22.67	90.91	81.37	<0.0001	0.8822-0.9582
	APE vs NSTEMI	0.9731	13.32	91.30	96.08	<0.0001	0.9507-0.9956
	APE vs AD	0.8782	17.56	80.77	91.18	<0.0001	0.7938-0.9626
	APE vs CPE	0.6880	39.70	88.57	50.00	0.0009	0.5974-0.7785
	Low+Intermediate-low vs Intermediate-high+High	0.8851	113.50	81.82	90.11	<0.0001	0.8030-0.9673
	Low+Intermediate-low vs CPE	0.6521	39.70	88.57	43.96	0.0083	0.5540-0.7503
	Intermediate-high+High vs CPE	0.9844	40.94	88.57	100.00	<0.0001	0.9564-1.000
GMO	APE vs Control	0.9529	30.55	93.31	87.25	<0.0001	0.9285-0.9772
	APE vs HC	0.9590	30.55	98.35	87.25	<0.0001	0.9312-0.9868
	APE vs NSTEMI	0.9665	18.65	91.30	95.10	<0.0001	0.9413-0.9918
	APE vs AD	0.8759	37.33	84.62	82.35	<0.0001	0.8029-0.9490
	APE vs CPE	0.7770	45.36	77.14	66.67	<0.0001	0.6979-0.8561
	Low+Intermediate-low vs Intermediate-high+High	0.8467	125.6	90.91	80.22	0.0002	0.7549-0.9384
	Low+Intermediate-low vs CPE	0.7516	45.36	77.14	62.64	<0.0001	0.6652-0.8381
	Intermediate-high+High vs CPE	0.9870	107.7	100.00	90.91	<0.0001	0.9587-1.000
GML+GMO	APE vs Control	0.9560	-	93.31	91.18	<0.0001	0.9330-0.9790
	APE vs HC	0.9600	-	99.17	87.25	<0.0001	0.9322-0.9878
	APE vs NSTEMI	0.9734	-	96.74	91.18	<0.0001	0.9509-0.9958
	APE vs AD	0.8876	-	80.77	88.24	<0.0001	0.8136-0.9617
	APE vs CPE	0.7810	-	77.14	68.63	<0.0001	0.7019-0.8600
	Low+Intermediate-low vs Intermediate-high+High	0.8811	-	81.82	86.81	<0.0001	0.8067-0.9556
	Low+Intermediate-low vs CPE	0.7576	-	65.71	75.82	<0.0001	0.6715-0.8437
	Intermediate-high+High vs CPE	0.9948	-	94.29	100.00	<0.0001	0.9811-1.000

Similarly, when differentiating APE patients from Control, HC, NSTEMI, and AD groups, GMO demonstrated AUC values of 0.9529, 0.9590, 0.9665, 0.8759, respectively. The sensitivities ranged from 91.30% to 98.35%, and specificities from 82.35% to 95.10%. (**Figure 5D**; **Table 3**). In addition, GMO exhibited an AUC of 0.7770 in discriminating APE from CPE, with sensitivity and specificity of 77.14% and 66.67%, respectively. (**Figure 5E**; **Table 3**). Furthermore, GMO displayed an AUC of 0.8467 in discriminating low-risk and intermediate-low-risk APE from intermediate-high-risk and high-risk APE, with sensitivity and specificity of 90.91% and 80.22%. Meanwhile, GMO revealed an AUC of 0.7516 in discriminating low-risk and intermediate-low-risk APE from CPE, and the optimal sensitivity and specificity were 77.14% and 62.64%, respectively. In discriminating intermediate-high-risk and high-risk APE from CPE, GMO revealed an AUC of 0.9870, with the optimal sensitivity and specificity of 100% and 90.91%, respectively (**Figure 5F**; **Table 3**).

Subsequently, we determined whether the combination of GML and GMO could improve the accurate diagnosis rate of APE patients. Logistic regression based on GML and GMO was used to construct a model. The logistic regression model for discriminating APE from control subjects (HC, NSTEMI, and AD) was: Logit(P) = -4.20 + 0.056×GML + 0.068×GMO. Both biomarkers contributed significantly to the model (GML: P = 0.023; GMO: P<0.001), achieving an AUC of 0.9560. As showed in **Figure 5G** and **Table 3**, the combination of GML and GMO demonstrated AUC values of 0.9560, 0.9600, 0.9734, 0.8876 in differentiating APE patients from Control, HC, NSTEMI, and AD groups, respectively. The sensitivities ranged from 80.77% to 99.17%, and specificities from 87.25% to 92.18%. In addition, the combination exhibited an AUC of 0.7810 in discriminating APE from CPE, with sensitivity and specificity of 77.14% and 68.63% (**Figure 5H**; **Table 3**). Furthermore, the combination displayed an AUC of 0.8811 in discriminating Low risk and Intermediate-low risk of APE from intermediate-high risk and high risk of APE, with sensitivity and specificity of 81.82% and 86.81%, respectively. Meanwhile, the combination revealed an AUC of 0.7576 in discriminating low risk and intermediate-low risk of APE from CPE, and the optimal sensitivity and specificity were 65.71% and 75.82%, respectively. The combination revealed an AUC of 0.9948 in discriminating intermediate-high risk and high risk of APE from CPE, with the optimal sensitivity and specificity of 94.29% and 100%, respectively (**Figure 5I**; **Table 3**). DeLong’s test was used to determine the statistical differences in diagnostic efficacy among GML, GMO and the combined model (**Supplementary Table 4**). The results indicated that there was no significant difference between the combined model and the individual indicators, which might be related to the strong correlation between GML and GMO (the r-value was 0.820, P<0.0001) (**Table 4**).

**Table 4:** Correlation Analysis of GML and GMO with Clinical Indicators.

Clinical Indicator	Statistics	GML(μg/mL)	GMO(μg/mL)
GML(μg/mL)	r	–	0.820
	P	–	<0.0001*
WBC(×10^9^/L)	r	0.213	0.150
	P	0.038*	0.146
PLT(×10^9^/L)	r	-0.154	-0.214
	P	0.136	0.036*
APTT(s)	r	-0.214	-0.129
	P	0.034*	0.203
FDP(μg/mL)	r	0.345	0.300
	P	0.0005***	0.003**
D-D(ng/mL)	r	0.322	0.300
	P	0.001**	0.003**

*P<0.05; **P<0.01; ***P<0.001.

### GML and GMO were significantly associated with clinical indicators in APE patients

As WBC, PLT, FDP, D-D and APTT has been reported to be dysregulated in APE (27–29), we next conducted the correlation analyses between GML, GMO and the clinical indicators in APE patients (**Table 4**). GML levels showed significant positive correlations with WBC, FDP and D-D (the r-values were 0.213, 0.345 and 0.322, respectively), while a negative correlation with APTT (the r-value was -0.214). Meanwhile, there was a slight negative correlation trend between GML and PLT (the r-value was -0.154, P>0.05). In addition, the level of GMO was significantly positively correlated with the level of FDP and D-D (the r-values were both 0.300), while significantly negatively correlated with the level of PLT (the r-value was -0.214). Meanwhile, GMO exhibited a slight positive correlation trend with WBC (the r-value was 0.150, P>0.05) and a slight negative correlation trend with APTT (the r-value was 0.203, P>0.05). Taken Together, these results suggested that serum GML and GMO were correlated with some clinical indicators in APE patients. Furthermore, to verify whether the diagnostic associations of GML and GMO were independent of baseline clinical characteristics, multivariable logistic regression analysis was performed adjusting for clinical covariates including inflammatory markers, coagulation parameters, hepatic/renal function, and lipid profiles. Both GML and GMO remained independently and significantly associated with APE diagnosis after adjustment, indicating that these biomarkers provide diagnostic information complementary to standard clinical parameters (**Table 5**).

**Table 5:** Multivariable logistic regression analysis of serum GML and GMO for diagnosis of acute pulmonary embolism.

Comparison	Biomarker univariate	OR (95% CI)	Adj. OR (95% CI)	*P-*value
APE vs HC	GML	1.226 (1.155-1.302)	1.420 (1.211-1.664)	<0.001
	GMO	1.223 (1.149-1.301)	1.556 (1.182-2.047)	0.002
APE vs NSTEMI	GML	1.226 (1.155-1.302)	1.285 (1.175-1.407)	<0.001
	GMO	1.044 (1.020-1.069)	1.017 (0.960-1.078)	0.561
APE vs AD	GML	1.050 (0.994-1.110)	1.022 (0.957-1.091)	0.523
	GMO	1.067 (1.032-1.103)	1.072 (1.033-1.112)	<0.001
APE vs CPE	GML	1.001 (0.987-1.016)	0.990 (0.958-1.022)	0.539
	GMO	1.033 (1.018-1.049)	1.030 (1.002-1.059)	0.035

Adj. OR, adjusted odds ratio.

### Decreased expression of GZMB, perforin, and granulysin in T cells of APE patients

**Figure 6:**
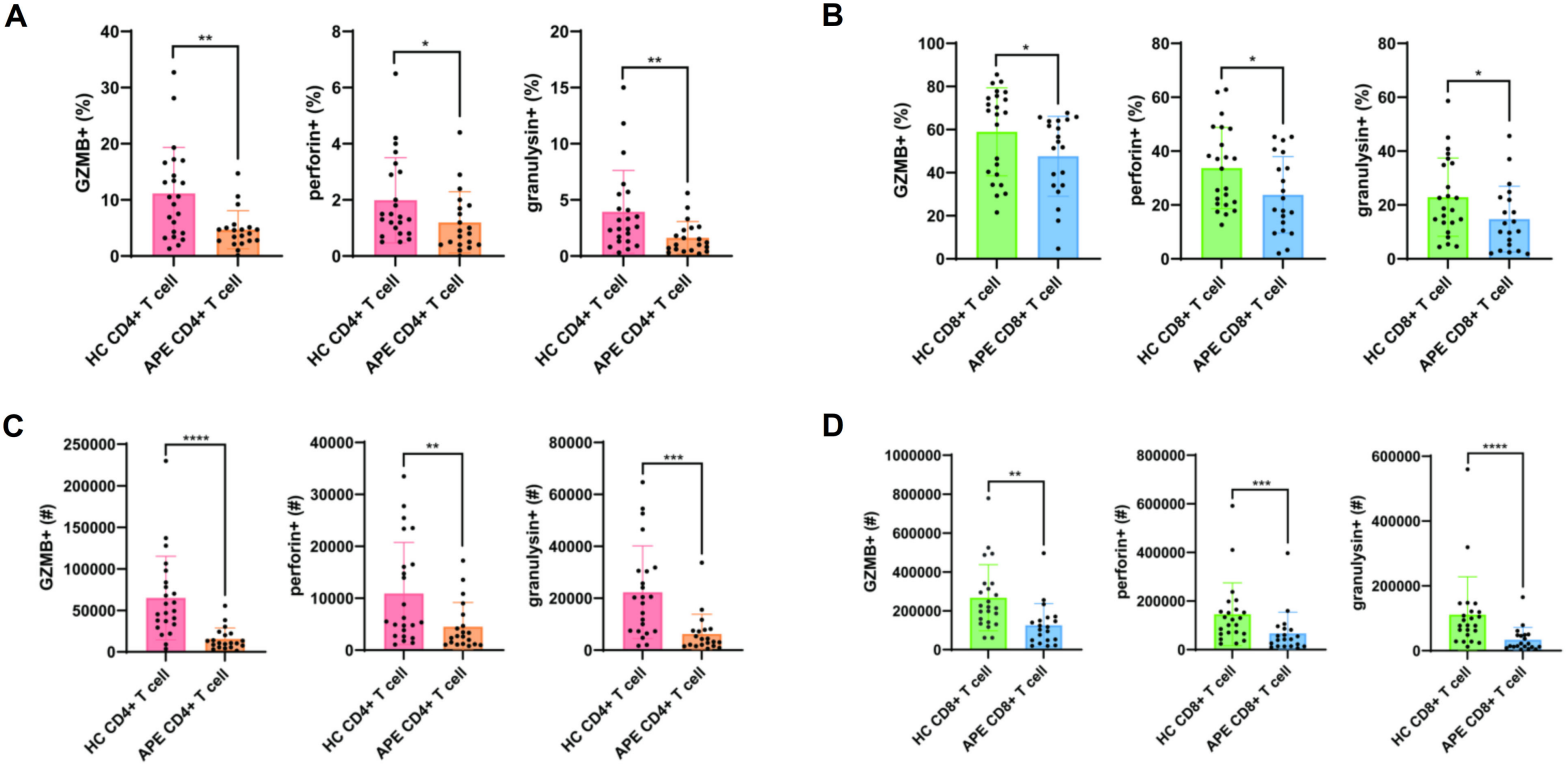
Expression levels of GZMB+, perforin+, and granulysin+ subsets in T cells between HC and APE. **(A)** Comparing the percentages of GZMB+, perforin+, and granulysin+ cell subsets within CD4+ T cells in peripheral blood between HC and APE patients. **(B)** Comparing the percentages of GZMB+, perforin+, and granulysin+ cell subsets within CD8+ T cells in peripheral blood between HC and APE patients. **(C)** Comparing the absolute cell counts of GZMB+, perforin+, and granulysin+ cell subsets within CD4+ T cells in peripheral blood between HC and APE patients. **(D)** Comparing the absolute cell counts of GZMB+, perforin+, and granulysin+ cell subsets within CD8+ T cells in peripheral blood between HC and APE patients. *P<0.05; **P<0.01; ***P<0.001; ****P<0.0001.

Previous studies have demonstrated that changes in the cytotoxic function of T cells played a critical role in the pathogenesis of cardiovascular diseases (30, 31). Next, we determined the changes in the cytotoxic function of T cells in APE patients. In order to analyze the cytotoxic function of T cells in patients with APE, we selected critical molecules associated with cytotoxic T lymphocytes (CTLs), including GZMB, perforin, and granulysin, and compared the differences of GZMB, perforin, and granulysin in T cells between HCs and APE patients. The results revealed that the percentage and the absolute numbers of GZMB+, perforin+, and granulysin+ in CD4+ T cells and CD8+ T cells were decreased in APE patients compared with HCs (**Figure 6**).

### GML and GMO reduce Notch1 expression in T cells

**Figure 7:**
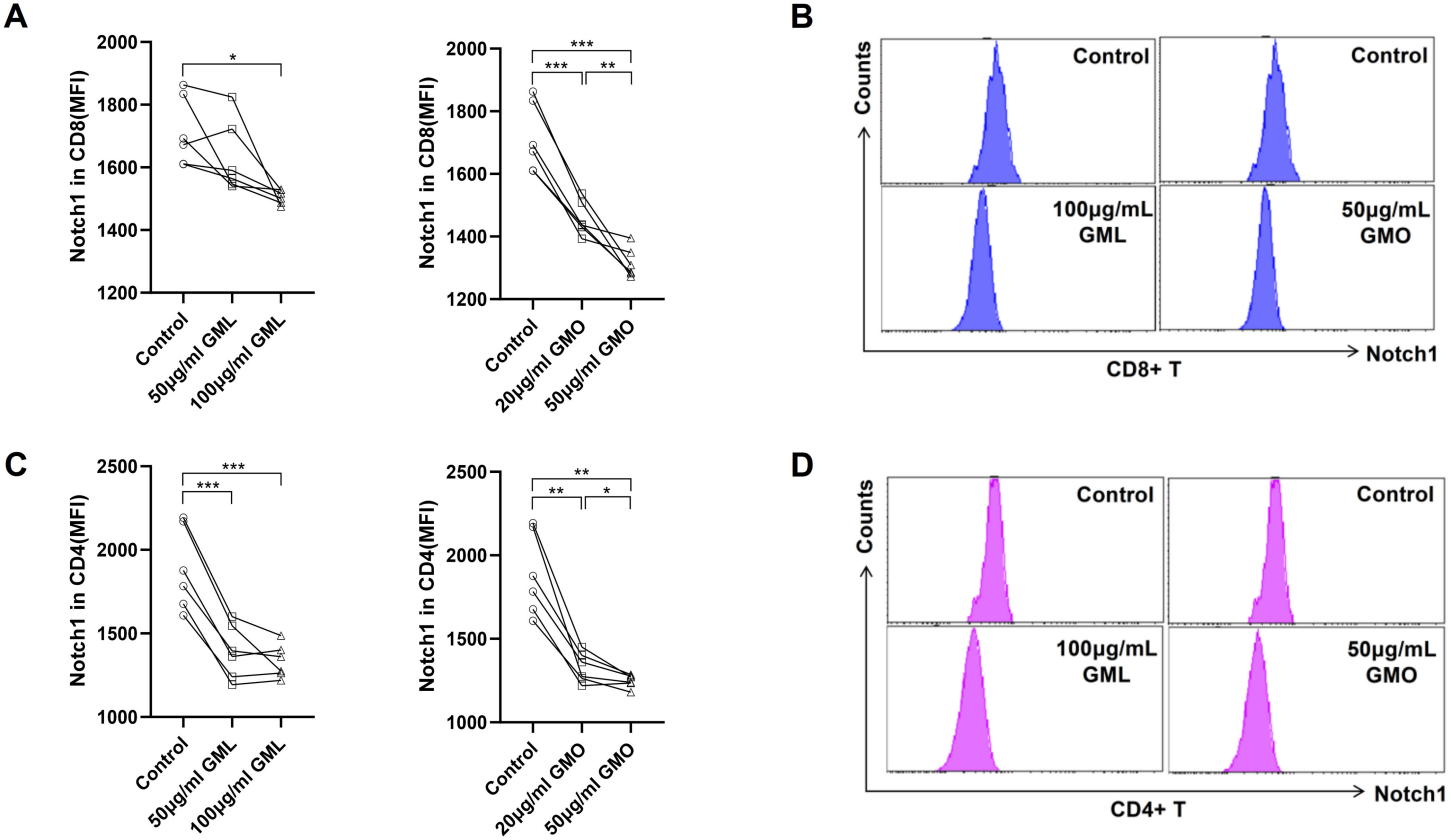
Notch1 expression levels in T cells after stimulation with GML and GMO. Each group was treated as follows: control, addition of 50 μg/mL GML or 20 μg/mL GMO, and addition of 100 μg/mL GML or 50 μg/mL GMO. **(A)** Comparing the effects of different concentrations of GML and GMO on Notch1 expression in CD8+ T cells. **(B)** Quantitatively assessing the impact of different concentrations of GML and GMO on Notch1 expression in CD8+ T cells by comparing histograms. **(C)** Comparing the effects of different concentrations of GML and GMO on Notch1 expression in CD4+ T cells. **(D)** Quantitatively assessing the impact of different concentrations of GML and GMO on Notch1 expression in CD4+ T cells by comparing histograms. *P<0.05; **P<0.01; ***P<0.001.

Notch1 signaling plays a critical role for the differentiation and effector functions of CD8+ T cells, and enhances the cytotoxic effect of CD8+ T cells by regulating the expression of transcription factors (such as T-bet and Eomes) and effector molecules (GZMB, perforin, and IFN - γ) (32). Therefore, we further explored whether GML and GMO could reduce the cytotoxic function of T cells by decreasing the expression of Notch1. As was shown in **Figure 7**, the mean fluorescence intensity (MFI) of Notch1 in the group treated with GML or GMO was significantly reduced compared with the unstimulated control group in CD8+ T cells. Similarly, both treatments significantly decreased Notch1 expression in CD4^+^ T cells. These results suggest that GML and GMO suppress Notch1 expression, which might contribute to the inhibition of T cell cytotoxic function.

### GML and GMO downregulate GZMB expression via Notch1 pathway inhibition

**Figure 8:**
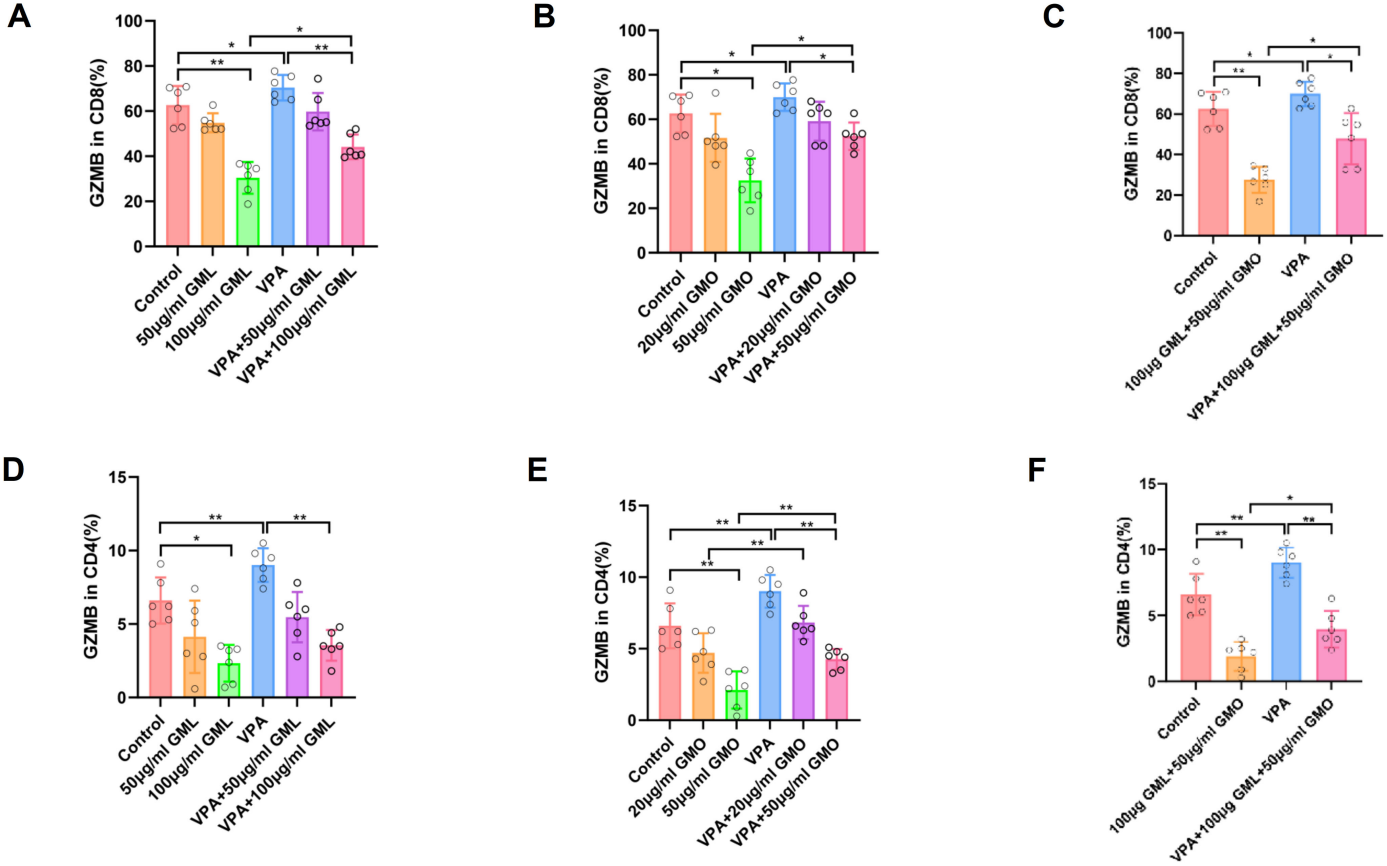
Expression levels of GZMB in T cells after stimulation with different concentrations of GML, GMO, and a Notch1 agonist. Each group was treated as follows: control, addition of 50 μg/mL GML or 20 μg/mL GMO, addition of 100 μg/mL GML or 50 μg/mL GMO, addition of 2 μM Notch1 signaling agonist VPA alone, addition of 2 μM VPA + 50 μg/mL GML or 2 μM VPA + 20 μg/mL GMO, and addition of 2 μM VPA + 100 μg/mL GML or 2 μM VPA + 50 μg/mL GMO. **(A)** Comparing the effects of different concentrations of GML and the Notch1 agonist VPA on GZMB expression in CD8+ T cells. **(B)** Comparing the effects of different concentrations of GMO and the Notch1 agonist VPA on GZMB expression in CD8+ T cells. **(C)** Assessing the impact of the simultaneous addition of GML, GMO, and the Notch1 agonist VPA on GZMB expression in CD8+ T cells. **(D)** Comparing the effects of different concentrations of GML and the Notch1 agonist VPA on GZMB expression in CD4+ T cells. **(E)** Comparing the effects of different concentrations of GMO and the Notch1 agonist VPA on GZMB expression in CD4+ T cells. **(F)** Assessing the impact of the simultaneous addition of GML, GMO, and the Notch1 agonist VPA on GZMB expression in CD4+ T cells. *P<0.05; **P<0.01.

As Notch1 signaling was critical for the cytotoxic effect of CD8+ T cells, we next assessed GZMB expression after GML and GMO treatment. Both GML and GMO significantly reduced GZMB levels in CD8^+^ and CD4^+^ T cells in a dose-dependent manner (**Figure 8**). To confirm the involvement of Notch1 signaling, we introduced sodium valproate (VPA), a Notch1 pathway activator. VPA markedly increased GZMB expression in cytotoxic T cells, whereas co-treatment with GML or GMO reversed this effect (**Figure 8**). These findings indicate that GML and GMO treatment downregulated surface Notch1 expression in cytotoxic T cells, which may contribute to the impaired CTLs function observed, thereby downregulating GZMB expression.

## Discussion

Our study demonstrates that elevated serum GML and GMO distinguish APE from HC, NSTEMI, and AD, and differentiate acute from chronic pulmonary embolism. These metabolites also correlate with risk stratification, suggesting utility in severity assessment. Mechanistically, the flow cytometry results demonstrated a decrease in the cytotoxicity of CTLs in APE patients, specifically manifested by reduced expression of molecules associated with cytotoxicity, such as GZMB, perforin, and granulysin. This decrease was associated with increased levels of GML and GMO in the peripheral blood serum of APE patients, and *in vitro* experiments showed these upregulated metabolites were associated with reduced Notch1 expression, suggesting a potential link to impaired cytotoxic function. The findings provided a new perspective for the early diagnosis and pathogenesis of APE.

Despite continuous advancements in global healthcare, APE remained one of the leading causes of death from cardiovascular diseases. In recent years, its incidence has been on an upward trend annually, imposing a significant burden on the healthcare system in our country (3). There was currently a lack of non-invasive biomarkers for APE with high sensitivity and specificity in clinical settings. Therefore, it is particularly important to seek novel biomarkers that can diagnose APE early and assess its prognosis. In this study, GML and GMO possessed good discriminative power in differentiating APE from HC, NSTEMI and AD groups with high sensitivity and specificity, showing great potential as diagnostic biomarkers for APE. Additionally, the levels of GML and GMO in the sera of high-risk APE patients were relatively higher than those of low-risk APE and CPE patients, indicating that GML and GMO had potential ancillary diagnostic value for risk stratification and prognosis assessment in APE patients. Furthermore, based on the results of DeLong’s test, we found that the diagnostic performance of the combined GML-GMO model showed no significant difference compared to the individual GML or GMO indicators. This lack of difference might be attributed to the strong correlation between GML and GMO. Besides GML and GMO, other lipids such as LPA(0:0/22:6), also showed significant differences, and might have potential diagnostic value as well. However, GML and GMO were prioritized because they exhibited the largest fold changes, and were centrally involved in glycerolipid metabolism. This study only focused on GML and GMO, and other lipids, such as LPA(0:0/22:6), should be further investigated to explore the broader metabolic landscape of APE pathogenesis in future studies.

Patients with APE suffer from a complex thrombo-inflammatory disease. Some studies had shown that in patients with APE, the white blood cell count was an independent predictor of short-term mortality and hospital readmission rates (33). Either an excessively high or low white blood cell count had an adverse impact on the progression and recovery of the disease. In addition, platelets might be activated and participated in the coagulation process, leading to a decrease in platelet count or changes in platelet function (34). Platelet count, platelet-related parameters, and other coagulation-related parameters were helpful for the assessment of thrombus burden, bleeding risk, and clinical prognosis (35). Fibrin degradation products (FDP) and D-dimer, as products of fibrinolysis, typically exhibited significantly elevated levels in patients with APE due to the processes of thrombus formation and dissolution (36–38). In our study, compared with the HC group, the APE group had significantly higher levels of WBC count, N%, PT, FDP, and D-D, and significantly lower levels of PLT. In the correlation analysis, it was found that serum GML and GMO were positively correlated with WBC, FDP and D-D, while negatively correlated with APTT and PLT, although some correlations were not statistically significant. Our results indicated that GML and GMO were closely associated with clinical indicators, and they might act synergistically to promote the disease progression of APE. In addition, the thrombi have become organized and the inflammatory milieu has shifted from an “acute storm” to a “smoldering” state dominated by sustained cytokines such as IL-6 and TNF-α in CPE, which might be associated with the modestly induce lipid reprogramming, but higher than those observed in diseases of a different nature, such as NSTEMI and AD (39). However, we recognize that D-dimer interpretation typically requires age-adjusted thresholds and clinical probability scoring, which were not incorporated in this analysis.

Long-chain fatty acids (LCFAs) exert bidirectional effects on T cell metabolism, differentiation, and function. It has been found that an enrichment of specific glycerophospholipids containing very-long-chain FAs (VLCFAs) could weaken the effector capacity of CTLs by affecting mitochondrial function (40). Linoleic acid might impair T cell function by inhibiting Th1/Th17 cell differentiation and cytokine production (41, 42), or induce T cells death (43). However, other studies have shown that linoleic acid could enhance CD8+ T cells metabolic fitness and antitumor immunity (21, 22). In addition, unsaturated fatty acid PE 18:1 could promote CTLs tumor infiltration via PPARγ/NF-κB/CCL5 pathway (44). Thus, LCFAs effect on T cells are context-dependent. The function of CD3+CD8+ cytotoxic T cells were significantly impaired in patients with pulmonary embolism, which affected the body’s ability to clear pathogens and tumor cells (45). Consistently, our study indicated that the cytotoxic capabilities of CTLs in individuals suffering from APE were decreased, and this decrease might primarily be attributed to the elevated levels of GML and GMO in APE patients.

Notch1 signaling regulates T cell differentiation and function, promoting CD4+ T cells polarization into Th1, Th2, or Tregs cells (46, 47) and enhancing CD8+ T cells cytotoixcity via transcription factors (such as T-bet, Eomes) and effector molecules (like GZMB, perforin, IFN-γ) (32). In this study, flow cytometry revealed a significant reduction in T cell cytotoxic function in APE, characterized by decreased expression of cytotoxic molecules such as GZMB, perforin, and granulysin. *In vitro* cell culture experiments demonstrated that GML and GMO treatment was associated with decreased Notch1 expression and reduced GZMB levels in T cells, suggesting these metabolites might contribute to impaired CTL function potentially through modulation of Notch1 related pathways.

This study had several limitations. At first, only 23 HC and 20 APE patients in the validation cohort were included in the flow cytometry assays, and the limited number tempered the strength of the direct inference between GMO/GML and immune function. In addition, this study is a small-scale, single-center study lacking an external validation cohort, and potential biases in patient selection and sampling might affect the external validity of GMO/GML and restrict the applicability of the research findings. For instance, the patients included in our study were predominantly low-risk APE cases, with relatively small number of intermediate-high-risk and high-risk patients. Although this sampling aligns with actual clinical scenarios, such bias might still impact the generalizability of our results. Moreover, in addition to cytotoxic T cells, the regulatory effects of GMO/GML on other cell types, such as Tregs, still require further investigation. It is important to note that our mechanistic findings are based primarily on expression-level evidence from *in vitro* experiments, in which we observed that GML and GMO treatment was associated with reduced Notch1 and GZMB expression, and definitive causal conclusions regarding GML/GMO as direct inhibitors of Notch1 signaling require further validation through genetic manipulation (e.g., Notch1 knockdown or overexpression) and detailed molecular interaction studies. In the future, synchronized multi-omics studies in large-scale and multicentered patient cohorts were required to establish their robustness across diverse populations.

In summary, this study was the first to confirm the upregulation of serum GML and GMO in APE, and both metabolites demonstrated good diagnostic performance in distinguishing APE from HC, NSTEMI, AD, and CPE patients. Additionally, the concentrations of GML and GMO in the serum of APE patients were associated with disease risk stratification, indicating that it might be useful for the prediction of patient prognosis. Moreover, GML and GMO in patients with APE appear to be associated with disease progression, potentially through a reduction in CTLs function linked to downregulated Notch1 expression; however, the precise mechanisms underlying this relationship remain unclear and warrant further investigation.

## Data Availability

The original contributions presented in the study are included in the article/**Supplementary Material**. Further inquiries can be directed to the corresponding author/s.
