# Supplementary figures and images for: Dysregulated lipid metabolites GML and GMO were associated with cytotoxic T cell function and serve as biomarkers for acute pulmonary embolism
Source: Front Immunol. 2026 Jul 8;17:1756977. doi: 10.3389/fimmu.2026.1756977 (PMC13388292; doi:10.3389/fimmu.2026.1756977)

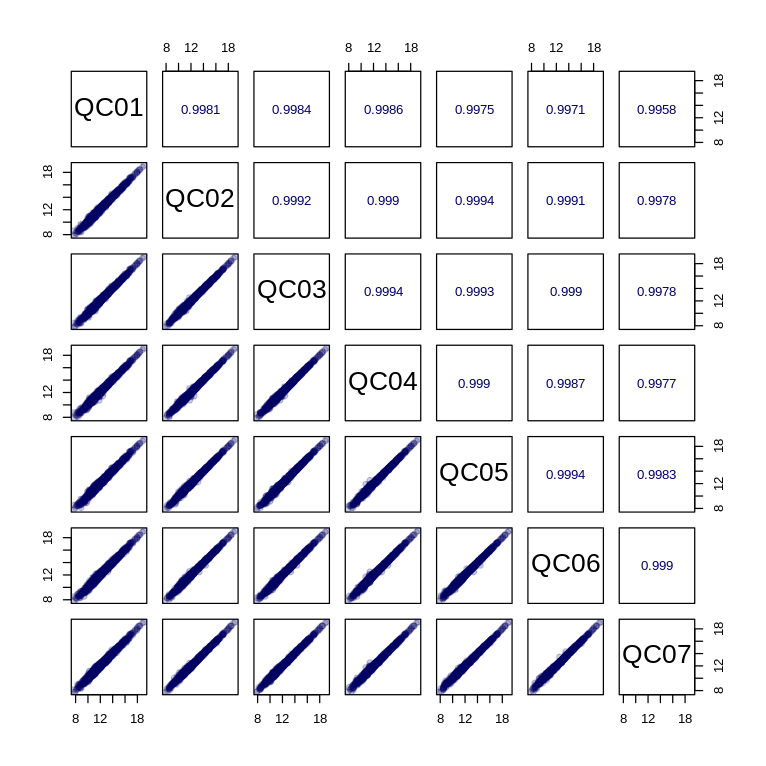

Supplement: Supplementary file 1 [file Image1.tif]

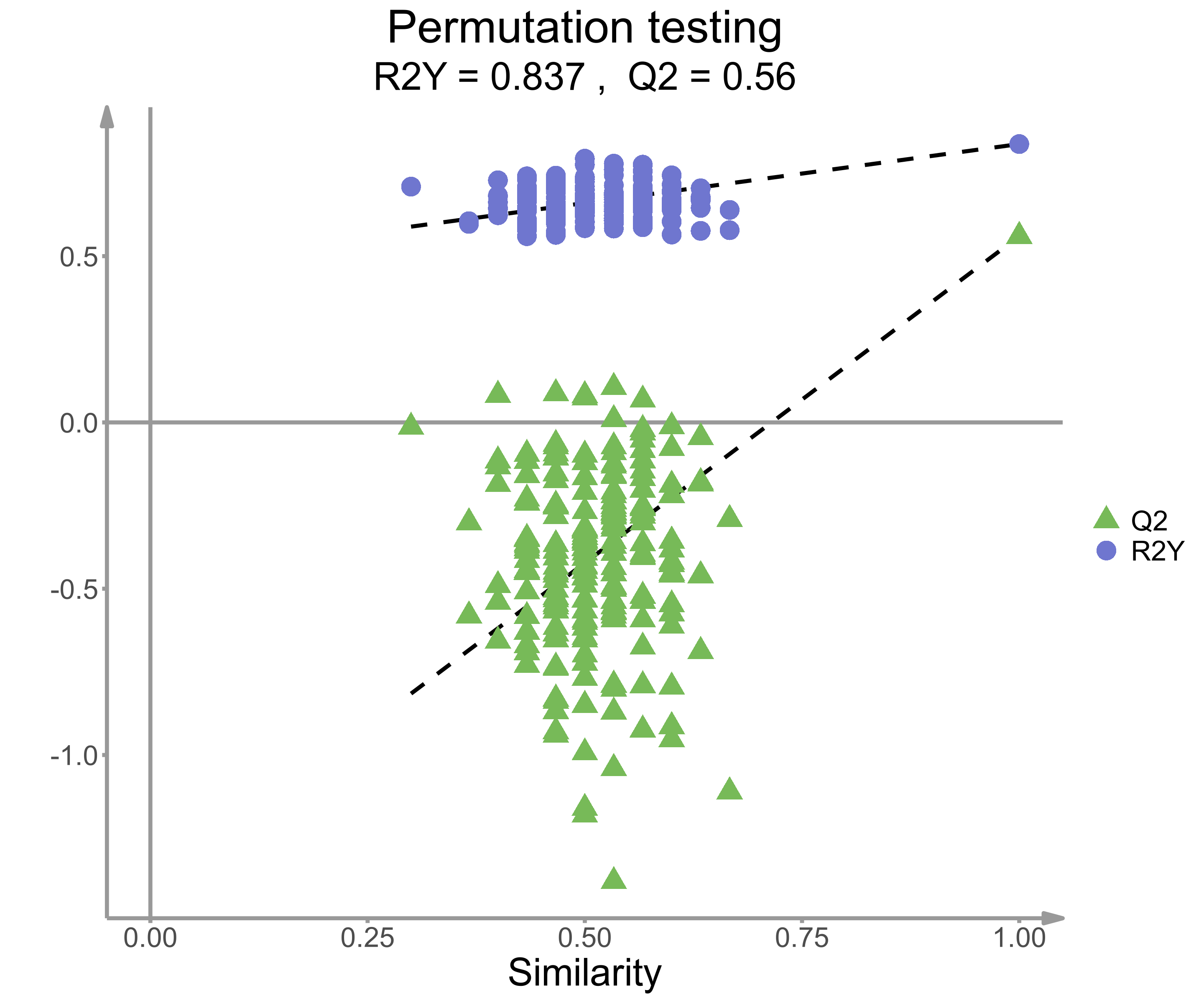

Supplement: Supplementary file 2 [file Image2.tiff]

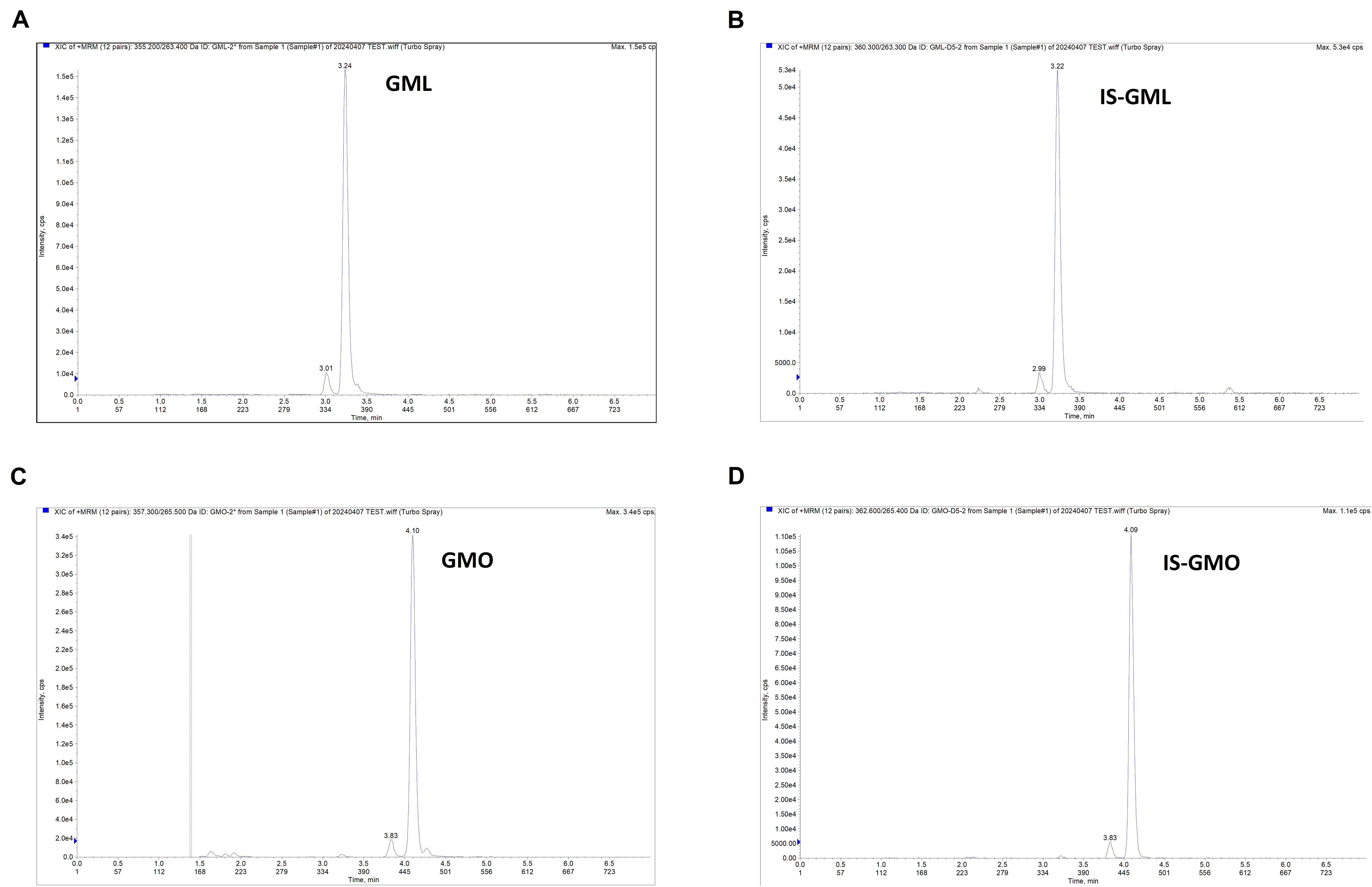

Supplement: Supplementary file 3 [file Image3.tif]

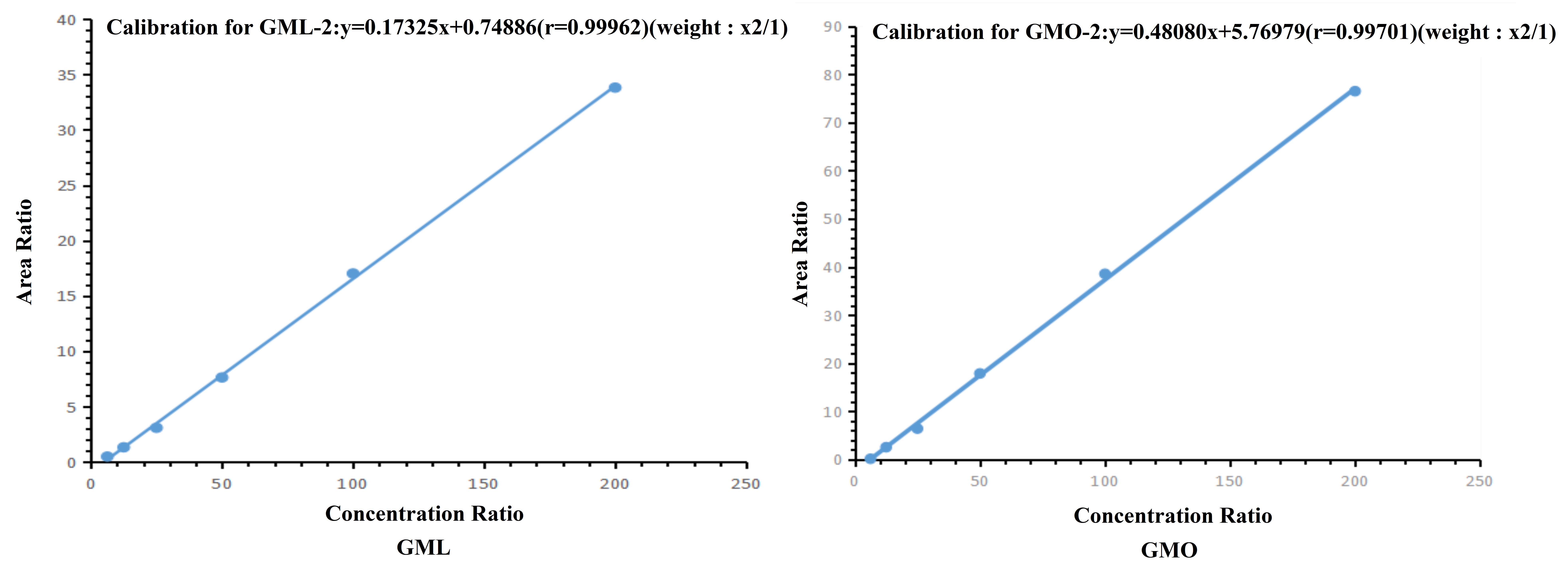

Supplement: Supplementary file 4 [file Image4.tif]

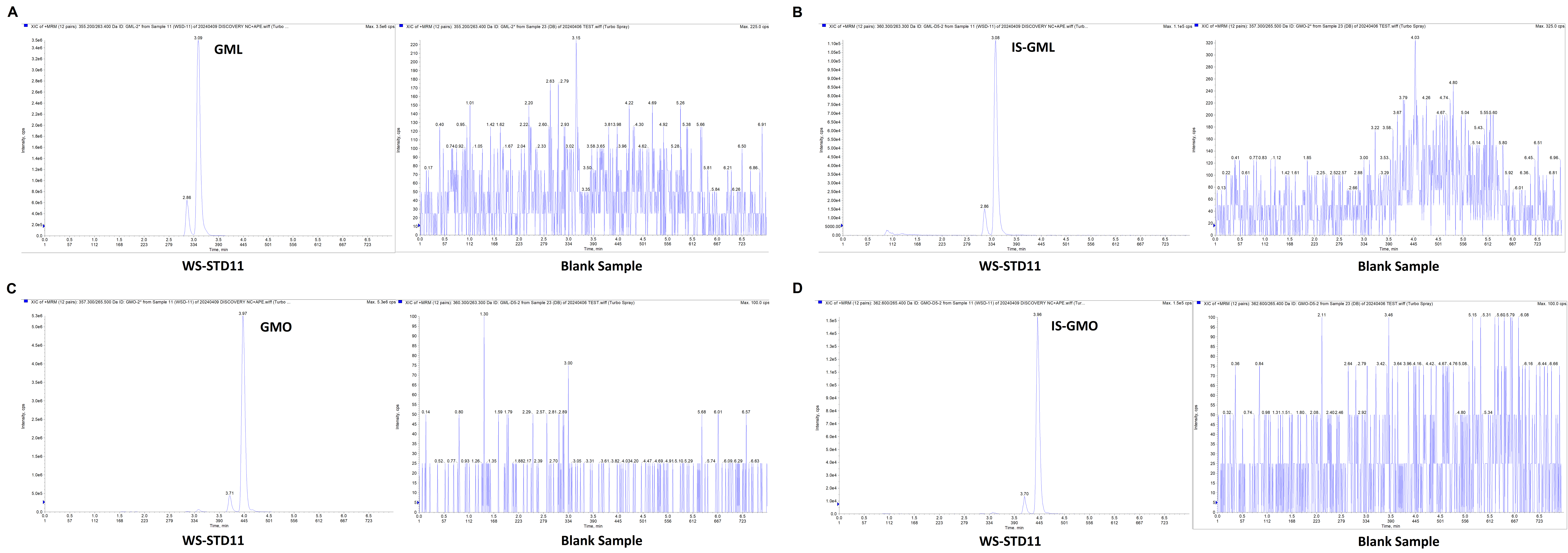

Supplement: Supplementary file 5 [file Image5.tif]
