# Supplementary material for: Dysregulated lipid metabolites GML and GMO were associated with cytotoxic T cell function and serve as biomarkers for acute pulmonary embolism
Source: Front Immunol. 2026 Jul 8;17:1756977. doi: 10.3389/fimmu.2026.1756977 (PMC13388292; doi:10.3389/fimmu.2026.1756977)
Supplement: Supplementary file 6 [file Table1.docx]

**Supplementary Table 1. The** **LC conditions and the gradient elution program of the LC-MS/MS method.**

| **Column** | C18,  2.6μm, 2.1*100mm | | **Column temperature** | | 40℃ | **Injection volume** | | 10μL | **Auto-sampler temperature** | | 2-8℃ |
| --- | --- | --- | --- | --- | --- | --- | --- | --- | --- | --- | --- |
| **Gradient elution program** | | | | | | | | | | | |
| **Time (min)** | | **Flow rate (ml/min)** | | **A%** | | | **B%** | | | **Switching valve** | |
| 0 | | 0.3 | | 15 | | | 85 | | | N/A | |
| 4 | | 0.3 | | 8 | | | 92 | | |  |  |
| 4.1 | | 0.3 | | 0 | | | 100 | | |  |  |
| 5.1 | | 0.3 | | 0 | | | 100 | | |  |  |
| 5.2 | | 0.3 | | 15 | | | 85 | | |  |  |
| 7 | | 0.3 | | 15 | | | 85 | | |  |  |
