# Supplementary material for: Dysregulated lipid metabolites GML and GMO were associated with cytotoxic T cell function and serve as biomarkers for acute pulmonary embolism
Source: Front Immunol. 2026 Jul 8;17:1756977. doi: 10.3389/fimmu.2026.1756977 (PMC13388292; doi:10.3389/fimmu.2026.1756977)
Supplement: Supplementary file 7 [file Table2.docx]

**Supplementary Table 2. The final multiple reaction monitoring (MRM) transitions of the LC-MS/MS method.**

| ID | Q1 Mass（Da） | Q3 Mass （Da） | Dwell  （msec） | CE  (volts) | DP  (volts) | EP  (volts) | CXP  (volts) |
| --- | --- | --- | --- | --- | --- | --- | --- |
| GML-1 | 355.2 | 338.4 | 40 | 15 | 40 | 10 | 6 |
| GML-2* | 355.2 | 263.4 | 40 | 15 | 80 | 10 | 6 |
| GML-3 | 355.2 | 245.4 | 40 | 18 | 80 | 10 | 6 |
| GML-D5-1 | 360.3 | 342.5 | 40 | 16 | 80 | 10 | 6 |
| GML-D5-2 | 360.3 | 263.3 | 40 | 16 | 80 | 10 | 6 |
| GML-D5-3 | 360.3 | 245.2 | 40 | 18 | 80 | 10 | 6 |
| GMO-D5-1 | 362.6 | 344.1 | 40 | 16 | 85 | 10 | 6 |
| GMO-D5-2 | 362.6 | 265.4 | 40 | 16 | 90 | 10 | 6 |
| GMO-D5-3 | 362.6 | 247.4 | 40 | 18 | 90 | 10 | 6 |
| GMO-1 | 357.3 | 339.4 | 40 | 15 | 85 | 10 | 6 |
| GMO-2 | 357.3 | 265.5 | 40 | 15 | 85 | 10 | 6 |
| GMO-3* | 357.3 | 247.1 | 40 | 18 | 85 | 10 | 6 |
