# Supplementary material for: Dysregulated lipid metabolites GML and GMO were associated with cytotoxic T cell function and serve as biomarkers for acute pulmonary embolism
Source: Front Immunol. 2026 Jul 8;17:1756977. doi: 10.3389/fimmu.2026.1756977 (PMC13388292; doi:10.3389/fimmu.2026.1756977)
Supplement: Supplementary file 9 [file Table4.docx]

**Supplementary Table 4. The statistical differences in diagnostic performance among the various models**

|  | Models | *P*-Value |
| --- | --- | --- |
| APE vs Control | AUC1 vs AUC2 | 0.1109^NS^ |
|  | AUC1 vs AUC3 | 0.0093^**^ |
|  | AUC2 vs AUC3 | 0.4357^NS^ |
| APE vs HC | AUC1 vs AUC2 | 0.0108^NS^ |
|  | AUC1 vs AUC3 | 0.0055^**^ |
|  | AUC2 vs AUC3 | 0.5774^NS^ |
| APE vs NSTEMI | AUC1 vs AUC2 | 0.2943^NS^ |
|  | AUC1 vs AUC3 | 0.7825^NS^ |
|  | AUC2 vs AUC3 | 0.2526^NS^ |
| APE vs AD | AUC1 vs AUC2 | 0.9443^NS^ |
|  | AUC1 vs AUC3 | 0.5719^NS^ |
|  | AUC2 vs AUC3 | 0.5184^NS^ |
| APE vs CPE | AUC1 vs AUC2 | 0.0107^*^ |
|  | AUC1 vs AUC3 | 0.0181^*^ |
|  | AUC2 vs AUC3 | 0.5597^NS^ |
| Low+Intermediate-low vs  Intermediate-high+High | AUC1 vs AUC2 | 0.3024^NS^ |
|  | AUC1 vs AUC3 | 0.8243^NS^ |
|  | AUC2 vs AUC3 | 0.1439^NS^ |
| Low+Intermediate-low  vs CPE | AUC1 vs AUC2 | 0.0098^**^ |
|  | AUC1 vs AUC3 | 0.027^*^ |
|  | AUC2 vs AUC3 | 0.6164^NS^ |
| Intermediate-high+High  vs CPE | AUC1 vs AUC2 | 0.8688^NS^ |
|  | AUC1 vs AUC3 | 0.3906^NS^ |
|  | AUC2 vs AUC3 | 0.383^NS^ |

NS, no significance, **P*＜0.05, ***P*＜0.01.
