# Supplementary material for: Dysregulated lipid metabolites GML and GMO were associated with cytotoxic T cell function and serve as biomarkers for acute pulmonary embolism
Source: Front Immunol. 2026 Jul 8;17:1756977. doi: 10.3389/fimmu.2026.1756977 (PMC13388292; doi:10.3389/fimmu.2026.1756977)
Supplement: Supplementary file 10 [file SupplementaryFile1.docx]

Supplementary Figure 1. The scatterplot matrix displayed the correlations between multiple quality control indicators (QC01 to QC07).

Supplementary Figure 2. The permutation test histogram demonstrating the correlation between variables, used to verify the predictive power of the model and the robustness of the relationships between variables.

Supplementary Figure 3. LC-MS/MS Chromatograms of GML and GMO. (A) The chromatogram of GML. (B) The chromatogram of IS-GML (Internal Standard for GML). (C) The chromatogram of GMO. (BD) The chromatogram of IS-GMO (Internal Standard for GMO).

Supplementary Figure 4. The calibration curves of GML and GMO. (A) The calibration curves of GML. (B) The calibration curves of GMO.

Supplementary Figure 5. The chromatograms of the substance peaks for the analytes GML and GMO, along with the internal standard peaks, at the highest point of the calibration curve, as well as those of the blank samples.
